# Synthesis and Comparative Study of the Structure and Antibacterial Activity of Polygalacturonate Complexes with Ionic and Nanoparticulate Silver

**DOI:** 10.3390/polym17202798

**Published:** 2025-10-20

**Authors:** Andrey V. Nemtarev, Elena V. Kuznetsova, Abdulla A. Yergeshov, Darya S. Eflova, Rezeda A. Ishkaeva, Inna R. Valiullina, Vladimir F. Mironov, Diana V. Salakhieva, Timur I. Abdullin

**Affiliations:** 1 Alexander Butlerov Institute of Chemistry, Kazan (Volga Region) Federal University, 18 Kremlyovskaya Street, 420008 Kazan, Russia; 2 Arbuzov Institute of Organic and Physical Chemistry, 8 Arbuzov Street, 420088 Kazan, Russia; 3 Institute of Fundamental Medicine and Biology, Kazan (Volga Region) Federal University, 18 Kremlyovskaya Street, 420008 Kazan, Russia; 4 Scientific and Educational Center of Pharmaceutics, Kazan (Volga Region) Federal University, 18 Kremlyovskaya Street, 420008 Kazan, Russia; 5 State Autonomous Healthcare Institution Republican Clinical Hospital of the Ministry of Health of the Republic of Tatarstan, 138 Orenburg Highway, 420064 Kazan, Russia

**Keywords:** sodium polygalacturonate, silver complex, Ag^+^, nanoparticles, antibacterial activity, drug resistance, cytotoxicity, structure-activity relationship, infected wound healing

## Abstract

A series of silver-polygalacturonate complexes with improved structure and activity against bacterial infections was developed. Pure sodium polygalacturonate was obtained by saponification of a pectin precursor and identified by NMR as predominantly homogalacturonan (uronide content 95%). Polygalacturonate complexes with ionic and borohydride-reduced silver with a controllable metallic component were synthesized; the role of spontaneous Ag^+^ reduction was revealed. The presence of uniform 5 nm nanoparticles and negligible particulate by-products in the reduced complexes was verified. The complexes showed similar silver-normalized activity against non-resistant bacteria, irrespective of complex stoichiometry/silver state. Pharmaceutical silver proteinate with a similar nanoparticle profile exhibited the same silver-normalized activity, indicating the lack of a ligand effect. The Ag^+^ complex was more effective against some hospital drug-resistant strains. The cytotoxicity of the complexes depended on fibroblast type, silver state, ligand type, exposure time, presumably in association with cellular availability and glutathione depletion. The complexes were administered to rats with excisional wounds persistently infected with *S. aureus*. Swab/histological analyses of the treated wounds revealed decreased bacterial burden/tissue damage, along with promotion of wound contraction/closure and matrix formation. The nanoparticle complexes that were compared had similar antibacterial/regenerative effects, while the Ag^+^ complex demonstrated higher efficacy in vivo. These results encourage the use of the developed silver-polygalacturonate complexes as antibacterial substances.

## 1. Introduction

Silver-containing complexes (SCs) in the form of ionic compounds and nanoparticles (NPs) represent important components of medicinal agents and medical materials, both clinically approved and being developed. The therapeutic effects of SCs are mainly directed against pathogenic microbial species and cancer cells [1]. The wide-spectrum antibacterial properties of SCs, along with the relatively low risk of resistance acquisition to them, are of permanent interest in antiseptic applications and treatment of infections caused by bacteria with different drug sensitivity profiles [2,3].

The silver ion (Ag^+^) is generally responsible for the antibacterial effects of SCs, including low-solubility compounds and NPs, which are present in equilibrium with Ag^+^ in aqueous media [3,4]. The accepted mechanisms of these effects involve immediate interaction of Ag^+^ with cell surface proteins, impairing their structure/activity and membrane integrity. Intracellularly, Ag^+^ interferes with metabolic processes by disturbing biomacromolecules, depleting antioxidants, and generating reactive oxygen species (ROS) [2,4,5]. In spite of the multiple biointeractions of Ag^+^, SCs exhibit relatively low toxicity upon both local and systemic administration [6,7,8], probably due to the lack of specific (e.g., cofactor) and direct prooxidant activities of Ag^+^.

In ionic complexes, Ag^+^ is bound to anionic low-molecular weight ligands (e.g., sulphadiazine and sulfathiazole) or macromolecular polyanionic ligands. The NP-containing complexes are commonly produced by reduction of Ag^+^ in the presence of a hydrophilic ligand that covers and stabilizes Ag^0^ colloids. Among ligands, natural and synthetic polymers often impart improved colloidal stability and pharmacokinetic properties to SCs [9,10] and facilitate their combination with medical materials and devices [11,12].

Protein ligands, which are widely used for this purpose, bind Ag^+^ via sulfhydryl, nitrogen-containing, and anionic groups [13,14,15]. Conversion of Ag^+^ to Ag^0^ and/or Ag_2_O NPs in the presence of a stabilizing protein underlies the formation of silver proteinates (colloidal silver proteins), which are of practical importance for local disinfection of mucosal membrane-lining organs [3,6,16], though they have limited medical application, such as treatment of nose and throat infections.

Polysaccharides are a promising alternative to potentially immunogenic protein ligands in terms of both chemical and biological characteristics. In particular, pectins containing α-1,4-linked galacturonic acid in the main macromolecular chain and neutral sugars in the side chains are abundant plant polysaccharides with important applications in the food industry, medicine, and pharmaceutics [17]. The established health-beneficial activities of pectin polysaccharides (PPSs) such as their immunomodulating, antioxidant, heavy metal-absorbing, and bacteriostatic properties [17,18], can supplement the specific effects of SCs. Furthermore, the gel-forming properties of PPSs are exploited for the development of drug-carrying/-eluting systems and tissue-engineered scaffolds [17].

Silver-PPS complexes can be readily obtained by association of anionic uronide groups with Ag^+^, which has a lower K_d_ than Na^+^ and K^+^ [19,20]. The intrinsic reducing and surface-active properties of PPS allow effective production of silver NPs upon heating of PPS-Ag^+^ complexes in solution [21]. The structural variability of PPSs obtained from different sources and under different conditions considerably affects the pharmaceutically relevant characteristics of the produced NPs, including their size, concentration, and activity [22,23]. A promising strategy to standardize the structure and properties of PPS is their de-esterification and partial hydrolysis to form polygalacturonic acid and its salts (polygalacturonates), which have increased solubility and structural homogeneity compared with pectins [24]. A series of polygalacturonate complexes with transition metals were proposed earlier [25,26,27,28,29,30]. The reported studies on SCs deal with polygalacturonate-mediated preparation of NPs [31] and composite wound dressing materials [11]. There is lack of polygalacturonate-based complexes with silver ions and NPs with proved antibacterial potential.

Our study focuses on the development of pharmaceutically relevant silver-polygalacturonate antibacterial substances with controllable polysaccharide ligand structure, as well as state and relative content of the silver component. The structure-antibacterial activity relationships of the complexes were assessed and compared between each other and with commercially available silver proteinate. The therapeutic potential of the developed substances was verified using an in vivo wound model with sustained bacterial infection. The obtained results provide an important basis for the production of improved silver-containing products for local antibacterial applications.

## 2. Materials and Methods

### 2.1. Materials

Citrus pectin (Herbstreith and Fox, Neuenbürg, Germany) and D-galacturonic acid (Acros Organics, Geel, Belgium) were used. Sodium borohydride, sodium tetraborate, hydrogen peroxide, sodium chlorite, sodium hydroxide, phenolphthalein, and silver nitrate were purchased from Acros Organics. Media, reagents, and consumables for cell culture and microbiology studies (DMEM, α-MEM, fetal bovine serum, Mueller-Hinton broth, bacteriological agar, etc.), as well as agarose for molecular biology and HEPES, were purchased from PanEco (Moscow, Russia). Resazurin, 2′,7′-dichlorodihydrofluorescin diacetate (DCFDA), and monochlorobimane were purchased from Sigma-Aldrich (St. Louis, MO, USA). Giemsa and picro-Mallory histological stains were obtained from BioVitrum (Saint Petersburg, Russia). Tiletamine–zolazepam (Virbac, Carros, France) and xylazine (Nita-Pharm, Saratov, Russia) were used for anesthesia. Ultrapure water (≥18.2 MΩ cm) was obtained with a Milli-Q^®^ Advantage A10 system (Merck Millipore, St. Louis, MO, USA).

### 2.2. Preparation of Silver-Polygalacturonate Complexes

#### 2.2.1. General

NMR spectra were recorded on a Bruker Avance-400 (^13^C-{^1^H}, 100.6 MHz) spectrometer (Bruker Daltonics, Billerica, MA, USA) at a temperature of 25 °C. FTIR spectra were registered on a Bruker Vector 22 spectrometer (Bruker Daltonics, Billerica, MA, USA) in KBr pellets. UV absorption spectra were registered on a UV-2600 spectrophotometer (Shimadzu, Kyoto, Japan). Optical rotation angles ([α]) were determined on a POL1/2 automatic compact polarimeter (Atago, Tokio, Japan). Organic elemental analysis was performed on a EuroEA 3028-HT-OM high-temperature CHNS-O analyzer (Eurovector SpA, Pavia, Italy). Metal content was determined on an iCAP 6300 DUO atomic emission spectrometer (ThermoFisher Scientific, Waltham, MA, USA). The conductivity and pH of test solutions were measured using a SevenDirect SD23 conductivity meter (Metler Toledo, Kusnacht, Switzerland) and InoLab pH7110 (WTW, Munich, Germany) pH meter, respectively. Dry polysaccharides were mechanically treated with a Vibrator DDR-GM9458 (NARVA, Brand-Erbisdorf, Germany) vibrational mill to obtain powder with a dispersity of ca. ≤ 125 µm.

#### 2.2.2. Characterization of Polysaccharides

The degree of esterification of polysaccharides was analyzed by titrimetric determination of carboxyl groups using a phenolphthalein indicator, as detailed in the Supplementary Materials. The uronide content of the polysaccharides was analyzed using the modified carbazole method and a D-galacturonic acid standard, as detailed in the Supplementary Materials.

The molecular weight (MW) of pectin was assessed by the viscometric method. A series of aqueous pectin solutions in 1% NaF in the concentration range 0.025–0.5% was prepared, and their relative viscosities (η) were analyzed using an Ostwald capillary viscometer (Labtex, Moscow, Russia) with a capillary diameter of 0.56 mm. The measurements were performed at 20 °C (*n* = 5). MW was calculated using the Mark-Kuhn-Hauwink equation, [η] = K × M^α^, where M is viscosity-average MW, η is intrinsic viscosity, K = 1.1 × 10^−5^, and α = 1.22 [24].

The MW of polygalacturonate was assessed by the colorimetric detection of the end groups with arsenomolybdate reagent, as detailed in the Supplementary Materials.

Residual moister was analyzed by the gravimetric method on an analytical balance. The samples were weighed before and after drying in an oven at a temperature of 105 °C until mass stabilization.

Critical aggregation concentration (CAC) was determined by the conductometric titration method. The conductivity of polysaccharide solutions in a concentration range 0.01–2% at a temperature of 20 °C was registered. The concentration dependence of the conductivity was plotted, and the CAC was determined at inflection points in linear relationships (Figure S1, Supplementary Materials).

#### 2.2.3. Preparation of Sodium Polygalacturonate

First, 5.0 g of pure pectin was dissolved in 500 mL of ultrapure water at a temperature of 40 °C. Then, the pectin solution was heated to 60 °C, and 150 mL of 0.1 N NaOH was added gradually (100 mL/min). The solution was stirred for 2 h, cooled to ambient temperature (RT), mixed with 1950 mL of ethanol, and kept at 5 °C for 12–16 h to precipitate polygalacturonate (sodium salt). The polygalacturonate was filtered, washed with 75% ethanol solution (2 × 250 mL), dried in air, and then dried under vacuum. Characteristics: yield 95%; moisture 5.5%; η 1.2454 mm^2^/c (20 °C, *c* 0.5, H_2_O). [α]^20^_D_ +226.2 (*c* 0.5, H_2_O). FTIR (ν, cm^−1^): 3432, 1610, 1422, 1331, 1239, 1148, 1101, 1015, 952. NMR ^13^C-{^1^H} (D_2_O, δ_C_ ppm, *J* Hz): 176.01 (C^6^, D-Gal*p*A), 104.94 (C^1^, D-Gal*p*), 99.52 (C^1^, D-Gal*p*A), 79.62 (C^2^, L-Rha*p*), 78.47 (C^4^, D-Gal*p*A), 78.25 (C^4^, D-Gal*p*), 75.09 (C^5^, D-Gal*p*), 73.89 (C^3^, D-Gal*p*), 72.41 (C^2^, D-Gal*p*), 71.87 (C^3^, D-Gal*p*A), 69.43 (C^2^, D-Gal*p*A), 68.70 (C^5^, D-Gal*p*A), 66.49 (C^5^, L-Rha*p*), 61.34 (C^6^, D-Gal*p*), 17.17 (C^6^, L-Rha*p*). Element content (%): C 33.42; H 5.08; Na 9.91.

#### 2.2.4. Oxidation of Polygalacturonate

First, 1.0 g of sodium polygalacturonate was dissolved in 100 mL of ultrapure water. Then, 7.2 mL of 15% H_2_O_2_ and 0.36 g of NaClO_2_ were sequentially added, and the mixture was stirred for 36 h at room temperature (RT). The product was precipitated with excess ethanol at +5 °C for 12–16 h. The precipitate was filtered, washed with 75% ethanol (2 × 50 mL), dried in air, and then dried under vacuum to obtain oxidized polygalacturonate. Characteristics: yield 94%; moisture 5.1%. FTIR (ν, cm^−1^): 3423, 1618, 1420, 1332, 1261, 1147, 1100, 1018, 952. NMR ^13^C-{^1^H} (D_2_O, δ_C_ ppm, *J* Hz): 175.95 (C^6^, D-Gal*p*A), 104.95 (C^1^, D-Gal*p*), 99.54 (C^1^, D-Gal*p*A), 79.62 (C^2^, L-Rha*p*), 78.48 (C^4^, D-Gal*p*A), 78.25 (C^4^, D-Gal*p*), 75.10 (C^5^, D-Gal*p*), 73.89 (C^3^, D-Gal*p*), 72.42 (C^2^, D-Gal*p*), 71.86 (C^3^, D-Gal*p*A), 69.43 (C^2^, D-Gal*p*A), 68.71 (C^5^, D-Gal*p*A), 66.49 (C^5^, L-Rha*p*), 61.34 (C^6^, D-Gal*p*), 17.17 (C^6^, L-Rha*p*). Element content (%): C 33.56; H 5.38; Na 9.97.

#### 2.2.5. Reduction of Polygalacturonate

First, 1.0 g of sodium polygalacturonate was dissolved in 100 mL of ultrapure water. Then, 0.3 g of NaBH_4_ was added, and the mixture was stirred for 24 h at RT. The product was precipitated with excess ethanol at +5 °C for 12–16 h. The precipitate was filtered, washed with 75% ethanol (2 × 50 mL), dried in air, and then dried under vacuum to obtain reduced polygalacturonate. Characteristics: yield 99%; moisture 2.1%. FTIR (ν, cm^−1^): 3448, 1618, 1420, 1332, 1261, 1100, 1017, 951. NMR ^13^C-{^1^H} (D_2_O, δ_C_ ppm, *J* Hz): 176.01 (C^6^, D-Gal*p*A), 104.95 (C^1^, D-Gal*p*), 99.52 (C^1^, D-Gal*p*A), 79.62 (C^2^, L-Rha*p*), 78.47 (C^4^, D-Gal*p*A), 78.25 (C^4^, D-Gal*p*), 75.10 (C^5^, D-Gal*p*), 73.89 (C^3^, D-Gal*p*), 72.42 (C^2^, D-Gal*p*), 71.88 (C^3^, D-Gal*p*A), 69.43 (C^2^, D-Gal*p*A), 68.70 (C^5^, D-Gal*p*A), 66.49 (C^5^, L-Rha*p*), 61.34 (C^6^, D-Gal*p*), 17.17 (C^6^, L-Rha*p*). Element content (%): C 34.62; H 5.26; Na 10.27.

#### 2.2.6. Preparation of Polygalacturonate Complexes with Ionic Silver

First, 1.0 g of sodium polygalacturonate was dissolved in 100 mL of ultrapure water. Then, an aliquot of a 0.02 M solution of AgNO_3_ was added, and the mixture was stirred at 40 °C for 2 h, followed by keeping it at 5 °C for 12 h. The product was precipitated with excess ethanol at +5 °C for 12 h, filtered, and dried under vacuum. By using different amounts of AgNO_3_, polygalacturonate complexes with different Na^+^:Ag^+^ molar ratios were obtained (compounds **1**–**4**, Table 1). Additionally, the complexes were prepared at the same Na^+^:Ag^+^ ratios but at other temperatures (RT and 60 °C); their characteristics are shown in the Supplementary Materials.

#### 2.2.7. Preparation of Polygalacturonate Complexes with Silver Nanoparticles

First, 1.0 g of compounds **1**–**4** was dissolved in 100 mL of ultrapure water. Then, an aliquot of a 1% solution of NaBH_4_ in 0.1 M sodium borate buffer (pH = 10) was added at a rate of 3 mL/min, and the mixture was stirred at 40 °C for 2 h. The resultant yellow solution was additionally kept at 5 °C for 12 h. The product was precipitated with excess ethanol, kept at +5 °C for 12 h, filtered, and dried under vacuum. By using polygalacturonate complexes with different Na^+^:Ag^+^ ratios and appropriate amounts of NaBH_4_, polygalacturonate-silver nanoparticle formulations were obtained (compounds **5**–**8**, Table 1).

### 2.3. Characterization of the Nanostructure of Silver-Polygalacturonate Complexes

#### 2.3.1. Dynamic Light Scattering

The hydrodynamic diameter (D*_H_*), zeta potential (ζ), and particle dispersion index (PDI) of polygalacturonate-silver formulations were analyzed using the dynamic light scattering (DLS) technique on a Zetasizer Nano ZS analyzer (Malvern Instruments, Malvern, UK) in DTS0012 and DTS1060 cuvettes at 25 °C. D*_H_* was determined according to Einstein−Stokes relation: D*_H_* = kBT/3phD_t_, where kB is the Boltzmann constant, T is the absolute temperature in Kelvin, h is the viscosity, and D_t_ is the translational diffusion coefficient. Freshly prepared solutions of the formulations in ultrapure water (1 mg/mL, pH = 7) were subjected to DLS analysis in triplicate. Additionally, the samples were analyzed after storage in the fridge.

#### 2.3.2. Transmission Electron Microscopy (TEM)

For TEM analysis, an aliquot (3–5 μL) of the formulations diluted in Milli-Q water was distributed on 3 mm formvar-carbon coated copper grids and dried at RT. The samples were analyzed on a Hitachi HT7700 Exalens microscope (Tokyo, Japan) at an accelerating voltage of 100 kV in high-contrast mode. The size of the silver nanoparticles detected in TEM images was calculated using ImageJ 1.48v software. Statistical analysis of size distribution in the groups was performed using the Data Analysis function in Microsoft Excel. The PDI was determined according to the following formula: PDI = (sd/µ)^2^, where sd is standard deviation and µ is mean value.

### 2.4. Study of Antibacterial Effects

#### 2.4.1. Minimal Inhibitory Concentrations

Laboratory strains of *S. aureus* ATCC 25923, *B. subtilis* ATCC 6633, *E. coli* ATCC 25922, and *K. pneumoniae* ATCC 13883 were used. To obtain overnight cultures, a bacterial inoculum was added to sterile Mueller-Hinton broth (MHB) and incubated overnight at 37 °C under constant shaking. The overnight culture was diluted with MHB to achieve an optical absorption of the bacterial suspension of 0.08–0.13 units (λ = 625 nm), which corresponds to 0.5 McFarland standard (∼1.5 × 10^8^ CFU/mL) [32]. The inhibitory activity of formulations toward bacteria growth was determined using the microdilution method in 96-well polystyrene plates. All stock solutions of the compounds for the in vitro study were prepared in ultrapure milli-Q grade water at a concentration of 20 mg/mL and sterilized using 0.22 µm syringe-driven filters. The compounds were serially diluted with MHB in a concentration range 0.009–4.8 mg/mL. The bacteria were cultured in the presence of compounds at a final density of ca. 1.5 × 10^6^ CFU/mL in a shaking incubator (35 °C, 180 rpm, 24 h). The minimal inhibitory concentrations (MICs) of the compounds were defined as the lowest concentrations at which bacterial growth was not observed.

#### 2.4.2. ζ and ROS Level of Bacterial Cells

For assessment of the ζ of bacteria, an overnight culture of *S. aureus* was diluted with phosphate-buffered saline (PBS, pH = 7.4) to a density of 1.5 × 10^8^ CFU/mL. The bacterial suspension was mixed with a formulation (1 mg/mL) and incubated at a temperature of 37 °C with agitation of 180 rpm for 2 h. The treated bacteria were centrifuged at 5000× *g* for 10 min, resuspended in 0.05 M HEPES buffer (pH = 7), and subjected to ζ analysis, as detailed in Section 2.3.1.

For assessment of ROS, the bacterial suspension (1.5 × 10^8^ CFU/mL) in PBS was pretreated with 20 µM DCFDA (37 °C, 180 rpm, 30 min) and centrifuged at 5000× *g* for 10 min. The bacterial pellets were resuspended in PBS at the initial density, transferred into 96-well plates, and mixed with the formulation (1 mg/mL). DCFDA fluorescence was detected using an Infinite 200 PRO microplate analyzer (Tecan) at λ_ex/em_ = 490/526 in ‘top’ mode. The mixture was maintained at 37 °C and 180 rpm between measurements.

#### 2.4.3. Study of Drug Susceptibility of Clinically Isolated Bacteria

Multidrug-resistant bacteria from the collection of the Republican Clinical Hospital (Kazan, Republic of Tatarstan, Russia) were used. Intrahospital bacterial strains were isolated and identified by MALDI-TOF mass spectrometry on a Biotyper Microflex system (Bruker Daltonics, Billerica, MA, USA). The drug susceptibility profiles of the isolated bacterial strains were determined by the disc diffusion method on Mueller-Hinton agar using antibiotic-loaded discs (Bio-Rad Laboratories, Hercules, CA, USA). The analysis was performed in accordance with EUCAST (European Committee on Antimicrobial Susceptibility Testing) recommendations, version 14.0. To assess the sensitivity of the bacteria toward the silver-containing complexes, the overnight culture pre-diluted to 0.5 McFarland standard was spread onto agarized medium in a 10 cm polystyrene Petri dish, followed by the addition of 10 µL solution aliquots of compounds **3**, **7**, and **9** normalized by silver content. The samples were incubated at a temperature of 35 °C for 18 h, followed by examination of the zone of bacterial growth inhibition. The bacteria were considered sensitive (S) if they were completely lysed in the inhibition zone, whereas the presence of individual colonies showed that the bacteria were resistant (R).

### 2.5. Assessment of Mammalian Cell Viability

#### 2.5.1. Cell Culture Conditions

NIH/3T3 mouse embryonic fibroblasts (ATCC CRL 1658) and early-passage human skin fibroblasts (HSF) were kindly provided by Dr. Ilnur Salafutdinov (Kazan Federal University). The cells were cultured aseptically in DMEM (3T3 cells) or α-MEM (HSF) containing 10% fetal bovine serum (FBS), 2 mM L-glutamine, 100 U/mL penicillin, and 100 μg/mL streptomycin under standard conditions in a CO_2_ incubator (temperature 37 °C, humidified air atmosphere with CO_2_ concentration maintained at 5%). The cells were grown in culture flasks to subconfluency and collected by treating with trypsin-EDTA dissociation solution.

#### 2.5.2. Viability Assay

The collected cells were seeded into 96-well plates at a density of 5.0 × 10^3^ or 2.5 × 10^3^ cells for 24 or 72 h culturing, respectively, and grown overnight under standard conditions. Then, the medium was replaced with fresh medium, and sterile compounds serially diluted with ultrapure water were added into the medium at a volume ratio of 1:9. The cells were cultured in the presence of the compounds for 24 or 72 h. Then, the medium was replaced with fresh medium supplemented with 70 µM resazurin. The cells were additionally cultured for 1.5 h, followed by fluorimetric detection of resorufin using an Infinite 200 PRO microplate analyzer (Tecan, Männedorf, Switzerland) at λ_ex/em_ = 540/590 nm. Cell viability, as measured through the optical signal, is presented as a percentage relative to the signal of untreated cells (100% viability).

#### 2.5.3. Detection of Cellular ROS and Glutathione

Pre-grown 3T3 cells were seeded into 96-well plates at a density of 1.2 × 10^4^ cells per well and cultured overnight. The cells were pre-stained with 5 µM DCFDA for 30 min, washed, and treated with polygalacturonate-silver formulations (1 mg/mL) or AgNO_3_ at an equivalent silver concentration. DCFDA fluorescence was detected using an Infinite 200 PRO microplate analyzer (Tecan, Männedorf, Switzerland) at λ_ex/em_ = 490/526 nm in ‘bottom’ mode.

For analysis of reduced glutathione (GSH), the cells were treated with the compounds under the same conditions as those used for ROS analysis. The cells were washed and stained with 5 µM monochlorobimane (MCB); the MCB fluorescence signal was detected in microplate format, as detailed earlier [33].

### 2.6. In Vivo Study

#### 2.6.1. Animals

Male Wistar rats (340 ± 38 g) were purchased from Biofarm Stezar (Vladimir, Russia, https://biopitomnik.ru/, accessed on 25 May 2023). Animal care was performed according to European regulations on the protection of experimental animals (Directive 2010/63/UE). The animals were housed at the recommended temperature and humidity conditions, with free access to water and full-ration certified feed. The in vivo study was reviewed and approved by the Institutional Animal Care Committee of the Kazan Federal University (protocol No. 22 from 26 June 2020).

#### 2.6.2. Infected Wound Model

The rats were anesthetized by intraperitoneal injection of tiletamine–zolazepam/xylazine (20–20/10 mg/kg). The animal skin in the dorsal area was shaved and cleansed with a 70% ethanol solution. The infected excisional wound model was implemented, as detailed earlier [34], with some modifications. Briefly, two full-skin round excisions ca. 12 mm in diameter and 2–3 mm in depth were made surgically. The distance between wounds was 2 cm. Silicone pads were mounted around the wound border and fixed using a sterile skin stapler ApposeTM ULC 35W (Covidien , Dublin, Ireland) to inhibit wound contraction. Each wound was washed with excess sterile isotonic solution (IS) prior to inoculation.

An inoculum of *S. aureus* (10^7^ CFU/mL) was prepared by dilution of an overnight culture of the bacteria with sterile IS and used immediately. An aliquot of the inoculum (100 µL) was dropped onto each wound followed by its covering with pre-cut sterile polyurethane film. During next 24 h, infection propagation occurred in the wounds. Compounds **3**, **7**, and **9** were solubilized in IS at a concentration of 20 mg/mL and sterilized using 0.22 µm syringe-driven filters. An aliquot of the freshly-prepared formulation (100 µL) was dropped onto the wound from one side of the animal; another wound was similarly treated with IS. The formulation/vehicle were applied alternately to right or left wounds on different animals. The treatment was repeated three times every 24 h; each time, the wounds were covered with sterile polyurethane film.

#### 2.6.3. Wound Swabbing

At day 7 post-infection, 100 µL of sterile IS was spread onto the wound, left in place for 1 min, and collected using a sterile cotton bud (Nuova Aptaca, Piedmont, Italy), which was used to gently swab the wound surface. The inoculate was immediately transferred into 2 mL of sterile PBS buffer and suspended with moderate shaking. An aliquot of the resultant suspension was spread onto Mueller-Hinton agar in a Petri dish and incubated for 48 h. The bacterial colonies that grew were counted, and the concentration of these colonies in the suspension (CFU/mL) is presented.

#### 2.6.4. Histological Analysis

The animals were sacrificed at day 7 and 14 post-infection, and the skin in the wound area was dissected, together with surrounding tissues. The explants were subjected to chemical fixation and treatment with organic solution and finally were embedded in paraffin medium, according to established recommendations [35]. The samples were cut into 7-µm-thick sections on a Microm HM355 microtome (ThermoFisher Scientific), subjected to histological staining [35], and visualized by bright-field microscopy under an Axio Observer Z1 microscope (Carl Zeiss, Oberkochen, Germany). The relative area of bacterial associates was assesses using ImageJ software, as detailed earlier [34,36].

### 2.7. Statistical Analysis

Data are presented as mean ± SD/SEM. Statistical significance was determined by one-way analysis of variance (ANOVA) followed by Tukey’s multiple comparison post-test (* *p* < 0.05, ** *p* < 0.01, *** *p* < 0.001).

## 3. Results

### 3.1. Characteristics of Polygalacturonate

**Scheme 1:**
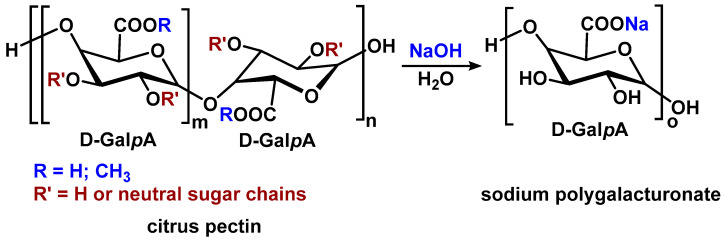
Preparation of sodium polygalacturonate (PGNa) from pectin.

Sodium polygalacturonate (poly-α-D-galactopyranosyluronate) was produced by means of alkaline saponification of citrus pectin at a temperature of 60 °C (Scheme 1). The product showed a profoundly increased uronide content (95 ± 1%) compared to that in pectin (73 ± 1%), and it was completely de-esterified, unlike the precursor, with degrees of esterification of 0 and 60 ± 1%, respectively.

**Figure 1:**
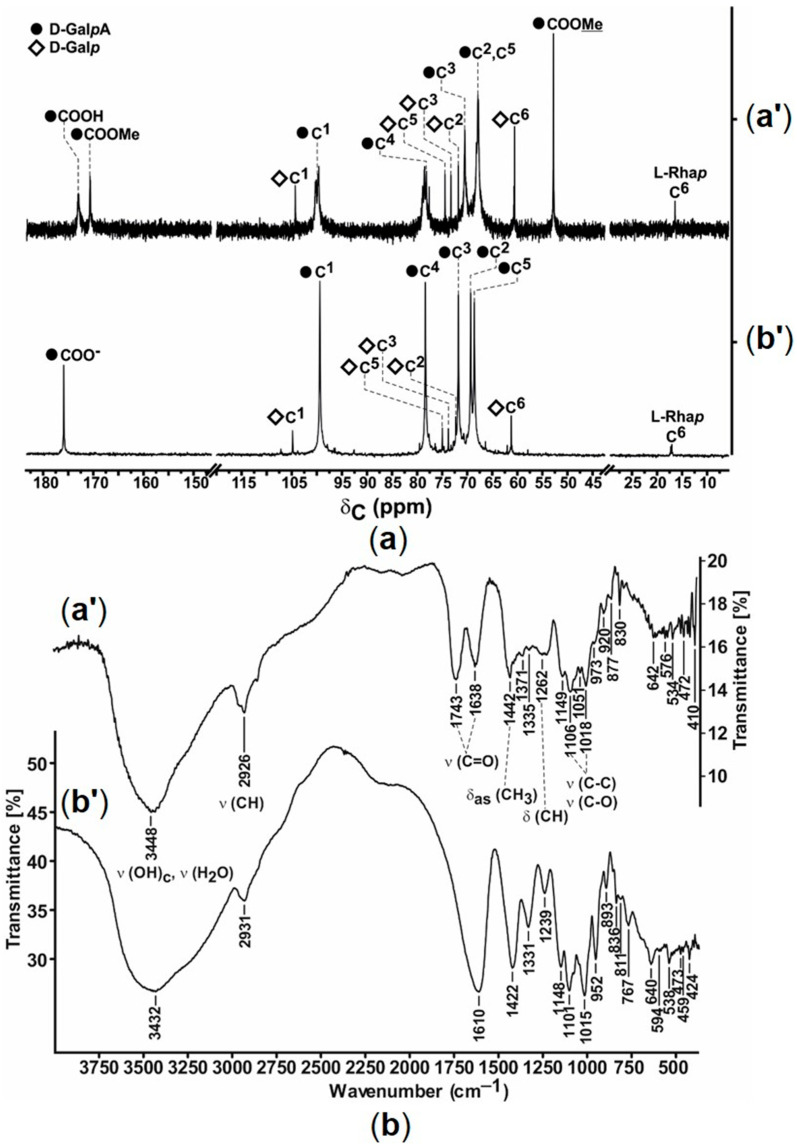
(**a**) ^13^C-{^1^H} NMR spectra of pectin (125.8 MHz, D_2_O) and sodium polygalacturonate (100.6 MHz, D_2_O) and (**b**) their FTIR spectra (KBr). Pectin and polygalacturonate are indicated as (a′) and (b′), respectively.

The ^13^C-{^1^H} NMR spectrum of pectin contained low-field signals at δ_C_ 173.08 and 170.77 ppm due to the free and methyl-esterified carboxyl groups of galacturonate fragments, respectively (Figure 1a). The signal at δ_C_ 16.54 ppm corresponded to the methyl groups of L-rhamnopyranose, indicating the rhamnogalacturonan nature of the polysaccharide. The presence of D-galactopyranose fragments was verified by the signals at δ_C_ 104.41 (C^1^), 77.71 (C^4^), 74.56 (C^5^), 73.37 (C^3^), 71.89 (C^2^), and 60.66 (C^6^) ppm. The ^13^C-{^1^H} NMR spectrum of polygalacturonate confirmed the disappearance of the carbon signals of the ester groups at δ_C_ 170 and 53 ppm. Furthermore, the signals from the neutral sugars, i.e., D-galactopyranose and L-rhamnopyranose, in polygalacturonate considerably decreased compared to those in pectin (Figure 1a), in accordance with the difference in uronide content.

The FTIR spectrum of pectin contained characteristic absorption bands at 1638 and 1743 cm^−1^, which corresponded to C=O bonds in free and methyl-esterified carboxyl groups, respectively. In the FTIR spectrum of polygalacturonate, the absorption band of the C=O bond appeared at 1619 cm^−1^, indicating ionization of the carboxyl groups of the polysaccharide (Figure 1b).

According to the conductometric method, pectin exhibited two critical aggregation concentrations (CACs) in aqueous solution at ca. 2.6 and 9.9 mg/mL (Figure S1a, Supplementary Materials). The lower CAC should correspond to a transition of single polysaccharide molecules into molecular associates, which further aggregate at higher CAC. Sodium polygalacturonate exhibited one critical aggregation concentration (CAC) in aqueous solution at ca. 4.1 mg/mL (Figure S1b, Supplementary Materials).

According to the viscometric method, 0.5% polygalacturonate solution had decreased viscosity compared to pectin (η = 3.1507 and 1.2454 mm^2^/s, respectively), so only the MW of pectin was determined by this method (29.7 ± 0.5 kDa; Section 2.2.2.), consistent with the other data for citrus pectins [37]. The lack of values for K and α constants in the Mark-Kuhn-Hauwink equation for polygalacturonate solutions did not allow determination of its MW. The end-group method based on the reduction of Cu^2+^ by terminal fragments of the polyuronide chain and colorimetric detection of the generated Cu^1+^ was used instead (Supplementary Materials). The MW of polygalacturonate assessed accordingly was 22.7 ± 0.1 kDa, suggesting some fragmentation of the starting polysaccharide.

In summary, with the use of pectin precursor, we synthesized pure sodium polygalacturonate, which was predominantly homogalacturonan (uronide content 95.0 ± 0.9%), with small amount of neutral sugars, i.e., D-galactose and L-rhamnose. Removal of side chains and ester groups in polygalacturonate increases its structural homogeneity and solubility in comparison with pectin. In addition, the produced polysaccharide should be more stable in aqueous solution, considering the increased hydrolytic stability of α-homogalacturonans compared with neutral α-glycans due to a different hydrolysis rate of the glycosidic bond between the anionic and neutral sugar fragments [38,39].

### 3.2. Composition of Silver-Polygalacturonate Complexes

**Scheme 2:**
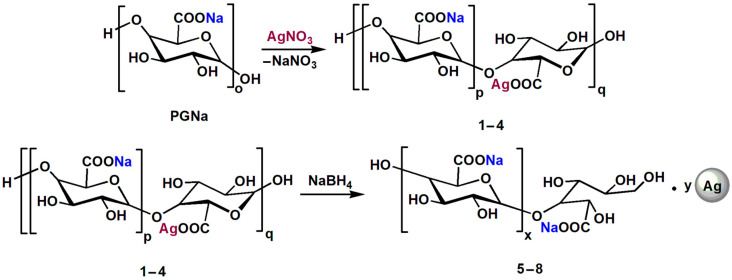
Preparation of sodium and silver polygalacturonate complexes with ionic silver (compounds **1**–**4**) and nanoparticulate silver (compounds **5**–**8**).

The ionic silver complexes were obtained by reaction of sodium polygalacturonate with AgNO_3_ at a given stoichiometry (Scheme 2). To designate the expected composition of the resultant mixed polygalacturonates (compounds **1**–**4**), we calculated the Na^+^:Ag^+^ molar ratio (Table 1), which was also the measure of the molar degree of substitution (DS) of sodium by silver (1–10%). The initial Na^+^ content in sodium polygalacturonate was determined by elemental analysis and atomic emission spectroscopy.

The experimental DS values determined by the elemental analysis (Table 1) were consistent with the calculated values for compounds **1**, **2**, **3**, and **4**, with theoretical DS values of 1, 2.5, 5, and 10%, respectively. The found mass contents of Ag in the mixed polygalacturonates were as follows: 0.49, 1.32, 2.63, and 5.18 wt.%. These data confirm effective inclusion of added Ag^+^ into the polygalacturonate backbone, in agreement with the high sorption capacity of PPS for transition metal ions [20]. The mixed polygalacturonates prepared at RT and 60 °C showed similar found silver contents to those for compounds **1**–**4** (Supplementary Materials).

**Figure 2:**
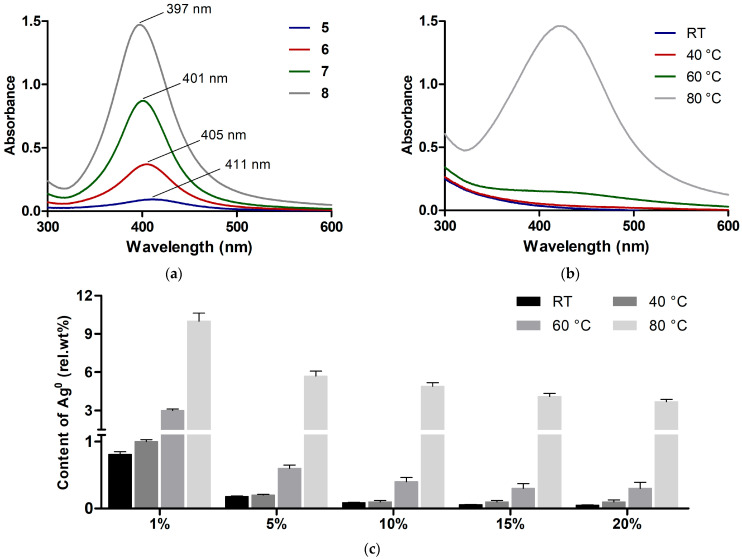
(**a**) UV-Vis absorption spectra of polygalacturonate complexes with silver nanoparticles **5**–**8** in aqueous solution (1 mg/mL). (**b**) UV-Vis absorption spectra of polygalacturonate complexes with ionic silver (DS = 5%) obtained at different temperatures (10 mg/mL aqueous solution). (**c**) Relative content of Ag^0^ (relative to total silver amount = 100%) in polygalacturonate complexes with different DS values.

By treating compounds **1**–**4** with NaBH_4_, ionic silver was converted to reduced (zero-valent) silver. UV-visible spectroscopy confirmed the formation of silver NPs in the products (compounds **5**–**8**, Scheme 2) in the proportion of DS, as revealed by characteristic plasmonic peaks with a maximum around 400 nm (Figure 2a).

The NP complexes **5**, **6**, **7**, and **8** were characterized by close Ag contents (0.50, 1.26, 2.48, and 5.06 wt.%) to those for the unreduced counterparts. Therefore, the formation of NPs does not change the stoichiometry of silver-polygalacturonate complexes. To additionally verify the structure of the synthesized compounds, IR spectroscopy analysis was carried out; the main IR signals were summarized in Table S1 (Supplementary Materials).

### 3.3. Intrinsic Reducing Ability of Polygalacturonate

The ability of polygalacturonate to reduce Ag^+^ was assessed to verify the homogeneity of the metallic component in both ionic and NP complexes. It was found that unreduced compounds **1**–**4** generated some plasmon resonance signals due to spontaneous reduction of Ag^+^ into Ag^0^ in the as-synthesized complexes.

As shown for compound **3**, with a DS value of 5%, at a temperature ≤ 40 °C the registered signal had a low intensity, and it greatly increased when the complex was obtained at 80 °C (Figure 2b), indicating heat-dependent Ag^+^ reduction presumably involving the terminal groups of polygalacturonate. The detected plasmonic peak was red-shifted compared to that detected for NaBH_4_-treated counterparts (Figure 2a), with λ_max_ values of 415–422 nm and 397–411 nm, respectively, indicating the formation of bigger NPs in the untreated ionic complexes. This is in agreement with the observation that the size of the generated NPs depends on the power of the reducing agent [21].

The existing relationship of λ_max_ and average size of silver NPs [40] allowed estimation of the size of NPs in the complexes **5**–**8**, which was less than 20 nm. Furthermore, by means of spectrophotometric detection of silver NP standards, the relative content of nanoparticulate silver (relative to total silver amount = 100%) in the Ag^+^-polygalacturonate complexes can be assessed.

In the unheated complexes (RT, 40 °C), the lower DS of 1% ensured higher relative conversion of Ag^+^ to Ag^0^, as if the reduction process was dependent on the ligand/metal ratio. For the compounds with DS > 1%, heating was required to achieve a significant amount of Ag^0^ (60 < 80 °C) (Figure 2b,c). Some hydrolytic depolymerization of polygalacturonate probably contributes to Ag^+^ reduction at elevated temperatures. These data show low spontaneous Ag^+^ reduction in complex with polygalacturonate at temperatures ≤ 40 °C.

**Scheme 3:**
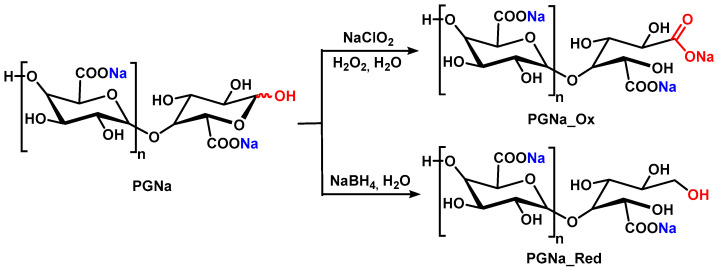
Oxidation and reduction of the terminal groups of sodium polygalacturonate.

**Figure 3:**
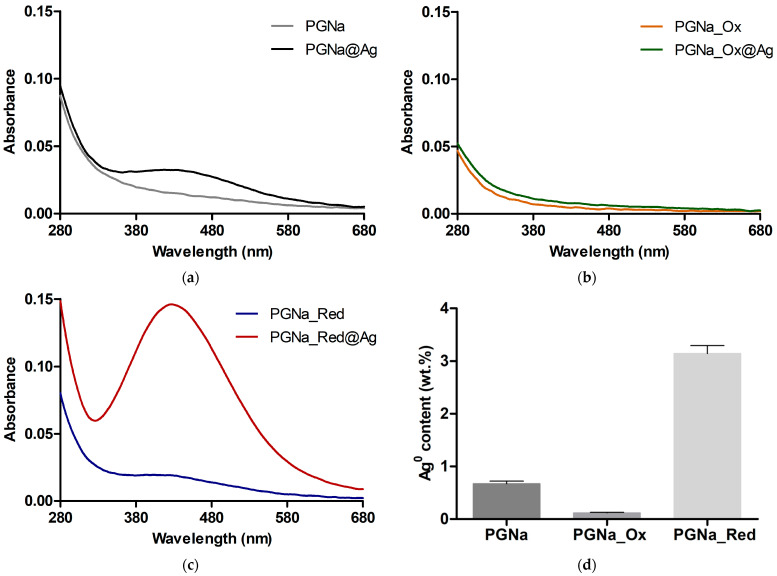
UV-Vis absorption spectra of silver-polygalacturonate complexes (DS = 10%) in aqueous solution (1 mg/mL) obtained from (**a**) initial polygalacturonate (PGNa), (**b**) pre-oxidized polygalacturonate (PGNa_Ox), and (**c**) pre-reduced polygalacturonate (PGNa_Red). (**d**) Relative content of Ag^0^ relative to total silver amount in the complexes (100%).

To additionally characterize the reducing capacity of polygalacturonate and the involvement of its end groups, it was pre-treated with sodium chlorite or borohydride to oxidize or reduce these groups into hydroxyhexanoic (PGNa_Red) and galactaric (PGNa_Ox) acids, respectively (Scheme 3). The resulting products were used to prepare complexes with Ag^+^. It was found that the oxidized polygalacturonate PGNa_Ox with a terminal galactaric acid fragment became non-reducing toward Ag^+^, whereas the reduced counterpart PGNa_Red showed an increased ability to generate Ag^0^ in comparison with initial polygalacturonate (Figure 3).

### 3.4. Association of Silver-Polygalacturonate Complexes in Solution

**Figure 4:**
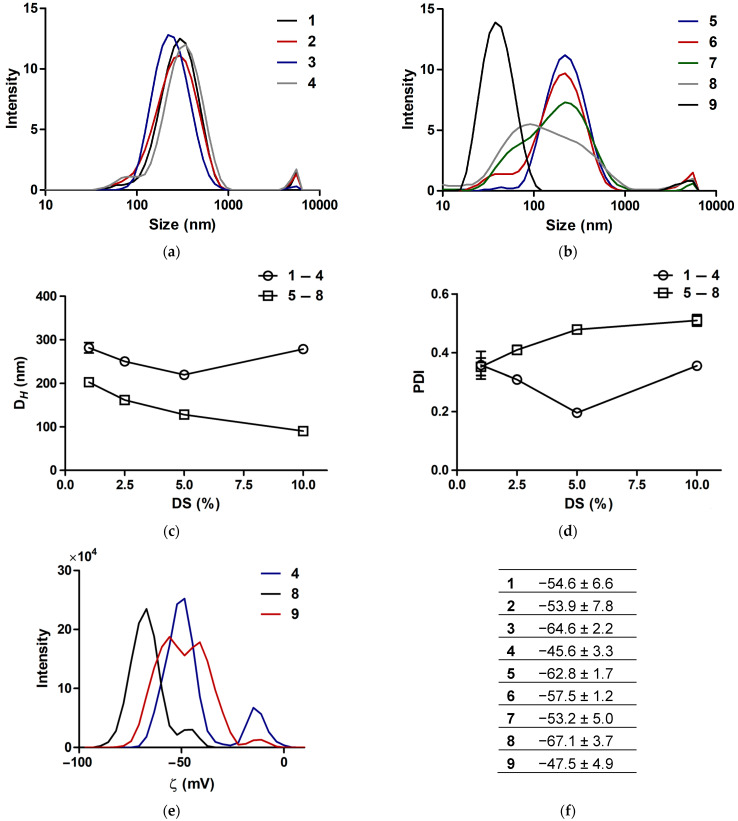
DLS data for silver-polygalacturonate complexes **1**–**8** and silver proteinate **9** in aqueous solution. (**a**,**b**) Distribution of hydrodynamic diameter. (**c**) Mean hydrodynamic diameter (Z-average) and (**d**) mean particle dispersion index. (**e**) Representative distribution curves and (**f**) mean values of zeta potential.

The DLS study showed that all the synthesized compounds **1**–**8** in aqueous solution generated well-defined peaks in the range between ca. 50 and 300 nm, attributed to intermolecular associates of polygalacturonate molecules rather than solid nanostructures (Figure 4). These associates had a high negative charge, with zeta potential (ζ) values from −45.6 to −67.1 mV, reflecting the polyanionic nature of polygalacturonate. In comparison with the Ag^+^-based complexes **1**–**4**, the Ag^0^-based counterparts **5**–**8** possessed smaller hydrodynamic diameters (D*_H_*) and higher particle dispersion indices (PDIs), presumably due to the effect of the NP component on the resultant DLS system (Figure 4). For complexes **1**–**4**, both D*_H_* and PDI gradually decreased with an increase of DS to 5%, suggesting compactization of the mixed polygalacturonates by Ag^+^. At an upper DS of 10%, however, the DLS system became bigger and more dispersed, presumably due to a relative decrease in the solubility of the molecules and promotion of their aggregation by the increased amount of Ag^+^. This was also accompanied by a noticeable diminution in the negative ζ of the associates (Figure 4).

Pharmaceutical-grade silver proteinate with a pre-verified silver content of 7.55 wt.% was additionally analyzed as reference compound **9**. It generated much smaller associates, with a D*_H_* of 37.7 nm and a PDI of 0.23. Presumably, this DLS system is determined by the NP component in the absence of the association of polypeptide ligand, in contrast to the polygalacturonate counterpart. Similarly to the polygalacturonate-based complexes, **9** exhibited anionic properties, with ζ of −47.5 mV (Figure 4). None of the compounds compared were impaired or collapsed upon storage in solution in a dark environment. Their D*_H_*/PDI values moderately and reversibly fluctuated during 21 days (Figure S2), supporting colloidal stability irrespective of the silver state/content and the ligand used.

### 3.5. Nanostructure of Silver-Polygalacturonate Complexes

**Figure 5:**
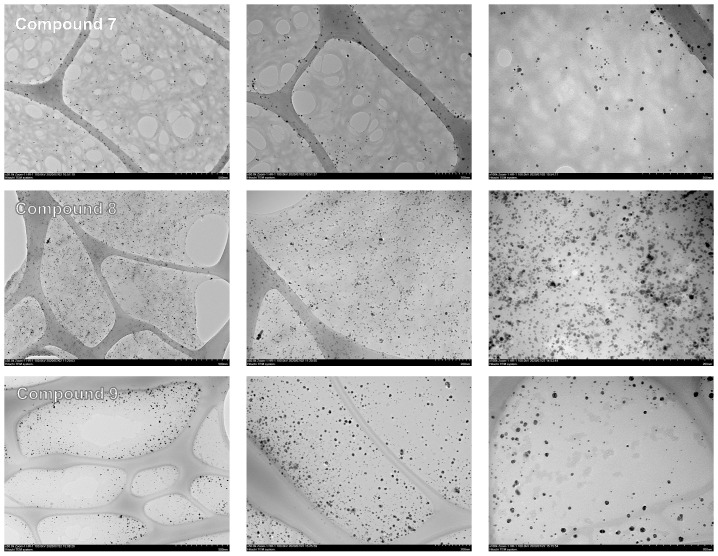
Representative TEM images of silver nanoparticle-containing complexes at increasing magnifications (from left to right).

**Figure 6:**
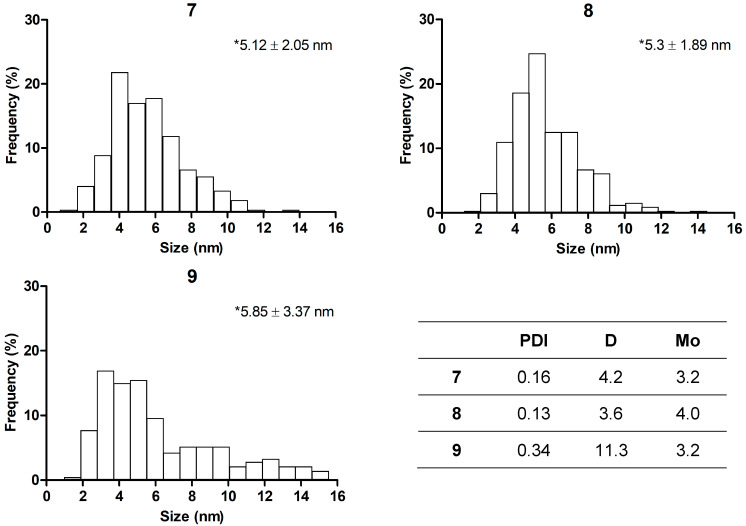
Distribution of nanoparticle size for compounds **7**, **8**, and **9**, according to TEM data. Asterisks show mean size ± SD. Insert: some homogeneity parameters, including PDI, dispersion (D), and mode (Mo).

The complexes with increased silver contents, namely, **3**, **4**, **7**, and **8**, as well as **9**, were subjected to TEM analysis. The reduced complexes **7** and **8**, along with **9**, and unlike their **3** and **4** ionic counterparts, contained homogeneous electron-dense NPs (Figure 5), confirming conversion of Ag^+^ into Ag^0^ colloids. **8** showed an increased NP amount compared with **7**, consistent with the initial DS of polygalacturonate. The size distributions of the detected NPs and their homogeneity parameters were analyzed (Figure 6). The mean sizes of the samples were quite similar between each other, namely, 5.1 nm (**7**) and 5.3 nm (**8**), and also to silver proteinate (5.9 nm). However, the NPs in **9** were more size-dispersed due to the presence of a bigger fraction (8–16 nm), which was not observed in the polygalacturonate complexes **7** and **8**. Under the same conditions, **3** and **4** contained rarely detected NPs, which were non-homogeneous and relatively big, i.e., up to tens of nanometers (Figure S3). These NPs were attributed to spontaneously generated NPs due to reduction of a small part of Ag^+^ by the polygalacturonate ligand, in agreement with spectrophotometric evaluation.

### 3.6. Antibacterial Activity

Table 2 shows the minimal inhibitory concentrations (MICs) of the studied compounds towards gram-positive and gram-negative bacteria. The corresponding MIC values decreased in the range from 2.4 to 0.1 mg/mL with increasing DS (silver content), without significant effect of the silver state (Ag^+^/Ag^0^) or the type of bacteria. Furthermore, when converted into absolute silver concentration, the mixed polygalacturonates **1**–**8** exhibited close activity to each other, which therefore depended on actual silver amount rather than the composition of the complexes. Moreover, in terms of silver amount, silver proteinate inhibited bacterial growth similarly to the polygalacturonates, suggesting the lack of a significant effect of the ligand’s nature. Both the mixed polygalacturonates **1**–**8** and **9** maintained their activity for at least 1 week of storage (Table S2), presumably in association with the colloidal stability of the compounds (Figure S2).

### 3.7. Effect on Cell Viability

**Figure 7:**
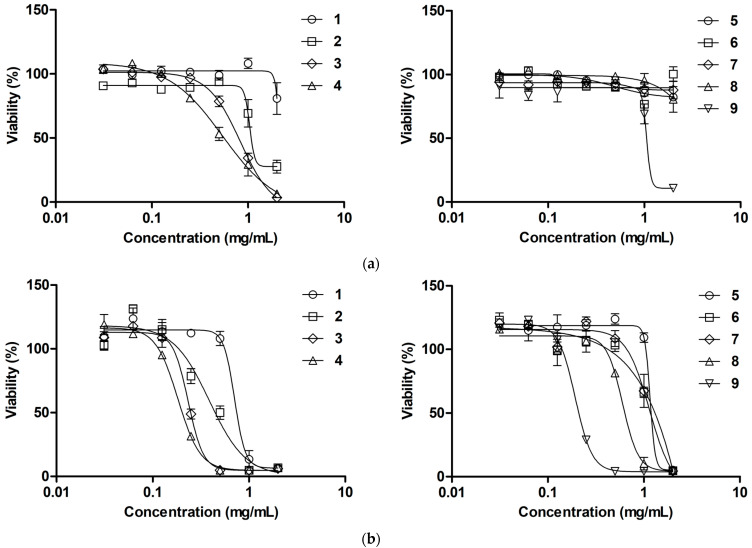
Concentration-dependent cytotoxicity of silver-containing compounds toward (**a**) 3T3 cells and (**b**) HSFs (resazurin assay, 24 h).

The effect of the complexes on the viability of mouse embryonic fibroblasts (3T3 cells) and primary human skin fibroblasts (HSF) after 24 and 72 h of culture was studied and compared (Figure 7, Table 3). Ag^0^-based compounds **5**–**8** did not exhibit noticeable cytotoxicity towards 3T3 cells when exposed for 24 h, while **9** showed some cytotoxicity at a concentration ≥1 mg/mL. Comparatively, Ag^+^-based counterparts **1**–**4** more strongly impaired cell viability in proportion to DS, though still with relatively high IC_50_ values (≥500 µg/mL). Similar relationships were revealed for HSFs, although these cells were more susceptible to the compounds, so that the silver content-dependent cytotoxicity was clearly observed (Figure 7).

Interestingly, after 72 h of treatment, the cytotoxicity of all the complexes was considerably (up to 26 times) increased toward 3T3 cells. In the case of HSFs, the complexes preserved a similar profile to that observed at 24 h treatment (Table 3, Figure S4). Although compounds **1**–**9** affected cell viability generally in proportion to silver content, this relationship was not straightforward, as if interaction of the complexes with mammalian cells was more intricate compared to that with bacterial cells. The found IC_50_ values of **1**–**8** (HSF, 24 h) were generally comparable to or higher than the corresponding MIC values, suggesting an acceptable difference in cytotoxic and antibacterial activities for local treatment of skin wounds.

### 3.8. Probing Compound-Cell Interactions

**Figure 8:**
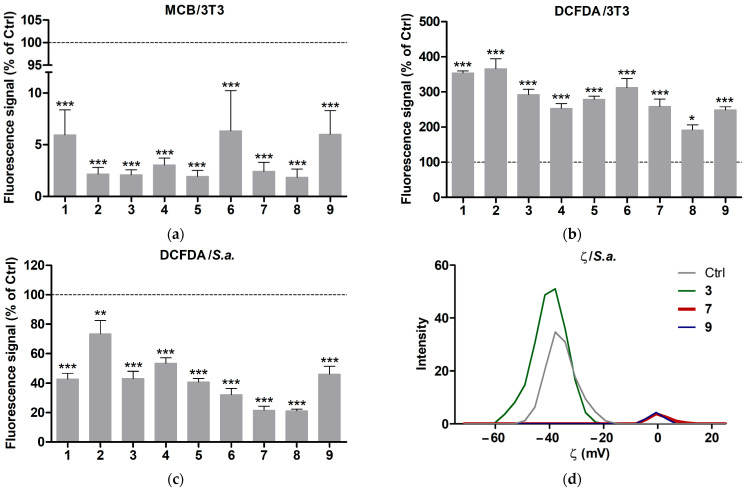
Effects of silver-containing compounds on (**a**) MCB and (**b**) DCFDA fluorescence signals in adhered 3T3 cells, as well as (**c**) the DCFDA signal and (**d**) the ζ of suspended *S. aureus* in buffer solution. The cells were treated with 1 mg/mL compounds for 2 h. The dashed line corresponds to the relative signal of untreated cells (100%). Means ± SEM (*** *p* < 0.001 vs. Ctrl) are shown for (**a**). Means ± SEM (* *p* < 0.05, ** *p* < 0.01, *** *p* < 0.001 vs. Ctrl) are shown for (**b**,**c**).

The effects of the compounds on intracellular levels of ROS and reduced glutathione (GSH), as well as on the ζ of suspended cells, were evaluated in buffer solution to avoid the protective action of medium components. According to MCB and DCFDA assays, 2 h treatment of 3T3 cells with **1**–**9** caused a profound decrease in GSH levels (Figure 8a) and moderate gradual increase in ROS levels (Figure 8b and Figure S5). This suggests that the compounds (their components) penetrate into the cells and cause drastic GSH depletion, apparently as a result of immediate reaction of the silver component with cellular thiols [41]. The concomitant ROS overproduction could arise from a deficiency of GSH, the major intracellular antioxidant, rather than the direct prooxidant action of Ag^+^. The ROS-modulating effect indicated higher activity of the Ag^+^-polygalacturonate complexes compared to their Ag^0^-based counterparts (Figure 8b), probably due to different availability/reactivity of the ionic and colloidal silver components. Furthermore, this effect tended to decrease with increasing silver content in the complexes, as if the relative silver amount affected the cellular availability of the mixed polygalacturonates under experimental conditions.

Unlike adhered 3T3 cells, suspended bacterial cells (*S. aureus*) showed a rapid decrease in their initial DCFDA signal (Figure 8c) followed by slow accumulation. This can be explained by compound-mediated aggregation of bacteria, probably in conjunction with interaction of the silver component with bacterial surface. Given this assumption, the reduced silver caused stronger bacterial aggregation than the ionic silver in the mixed polygalacturonates (Figure 8c).

According to the DLS analysis, bacteria treated with the selected compounds **3**, **7**, and **9** strongly affected cell ζ in different manners. **3** tended to increase the average negative charge of the bacterial suspension from −35.5 ± 1.2 to −37.4 ± 0.9 mV, along with some increase in DLS signal intensity, whereas exposure to both **7** and **9** resulted in an apparent loss of cell surface charge, along with a drop in the signal intensity (Figure 8d). These data and the above data suggest that the compounds studied interact with the bacterial surface, promoting aggregation of suspended bacteria to different extents (Ag^0^ > Ag^+^). Together, the results demonstrate that probing cellular antioxidants and ζ can provide useful information regarding interactions of silver-containing products with living cells.

### 3.9. Effects of Silver-Containing Compounds on Infected Wounds 

#### 3.9.1. Visual Examination and Swab Assay Data

**Figure 9:**
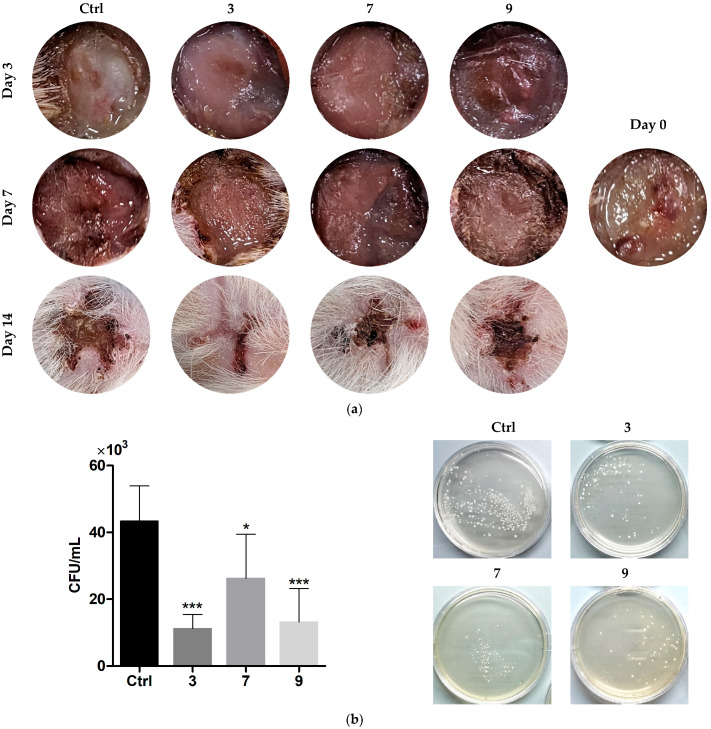
(**a**) Pictures of infected wounds after treatment with silver-containing compounds over 14 days. (**b**) Bacterial content of the treated wounds, according to swab analysis at day 7. Means ± SD are shown (* *p* > 0.05, *** *p* > 0.001 vs. Ctrl). The inset in (**a**) shows the wound post-infection (day 0).

The in vivo model involved formation of excisional wounds in rats and their inoculation with *S. aureus*, as described earlier [34]. The next day post-infection, repetitive topical therapy with solutions of selected compounds **3**, **7**, and **9** (20 mg/mL in isotonic solution) was begun. Visual analysis of the wounds confirmed the purulent process, which was relatively weaker in the compound-treated groups compared to the untreated control (Figure 9).

Swab analysis at day 7 showed a significant reduction in bacterial load in the treated wounds. Compounds **3** and **9** exhibited greater effects than **7**, with CFU decreasing factors of ca. 3.9 (**3**, **9**) and 1.3 (**7**), respectively. The wounds started to close to different extents during the observational period, with a somewhat higher effect of **3** compared to the other compounds.

#### 3.9.2. Histological Data

**Figure 10:**
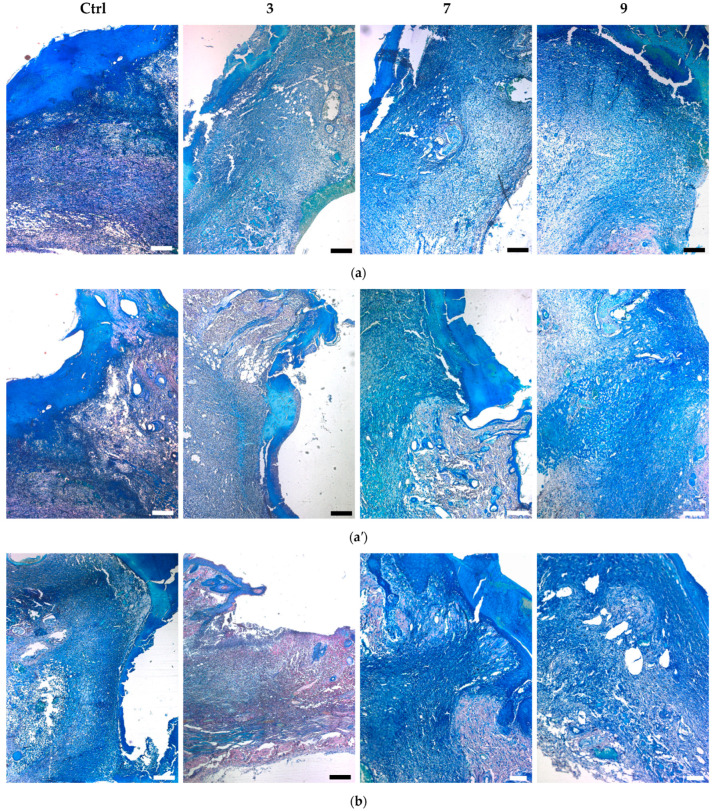

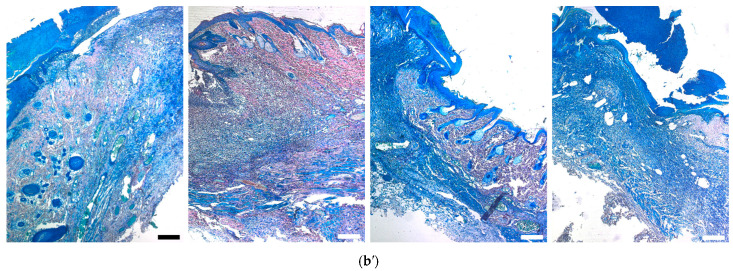
Giemsa-stained sections of infected wounds treated with silver-containing compounds (**a**,**a**′) at day 7 and (**b**,**b**′) at day 14. (**a**,**b**) and (**a**′,**b**′) show center and edge wound areas, respectively. Scale bars are 500 µm (except **7** and **9** in (**b**), with scale bars of 200 µm).

**Figure 11:**
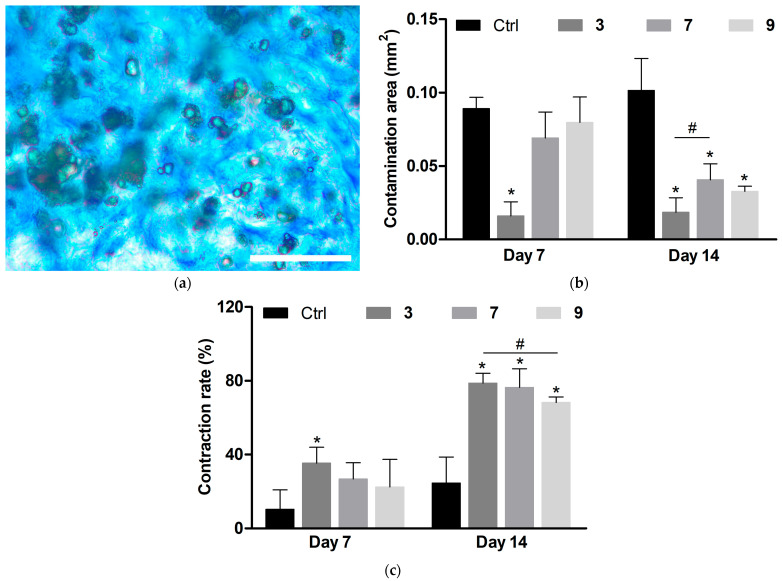
Quantitative data of histological analysis of infected wounds treated with silver-containing compounds. (**a**) Representative bright-field microscopy image of bacterial associates in wound tissues at increased magnification (scale bar is 20 µm). (**b**) Area occupied by bacterial associates per section. (**c**) Wound contraction rate. Means ± SD are shown (* *p* < 0.05 vs. Ctrl; # *p* < 0.05). Ctrl indicates untreated wounds.

Histological assessment of middle cross-sections of wound tissues at 7 and 14 days involved evaluation of the relative area occupied by bacteria, bacterial penetration into adjacent skin tissues, detritus formation, collagenization, and wound contraction. In Giemsa-stained sections, bacteria were detected as small dark dots (Figure 10), which at increased magnification appeared as greenish cells (Figure 11a). The contamination area (CA) per section was calculated using ImageJ software by specific color associated with bacteria [34,36].

At day 7, all groups with applied compounds showed lower wound infiltration by bacteria compared to the control group (Figure 10a). The decreasing effect of compound **3** on CA was higher than that of compounds **7** and **9** (Figure 11b).

At day 14, CA in the untreated wounds did not diminish (Figure 11b), indicating persistence of the infectious process, which is typical of chronically infected wounds. As previously shown, acute wounds in rats normally cope with bacterial inoculate and start to heal by this period [34]. The applied compounds further suppressed contamination compared to day 7, although the treated wounds still contained significant amount of bacteria (Figure 11b).

In proportion to wound contamination, bacteria penetrated into surrounding skin tissues, resulting in significant damage to the dermal, epidermal, and subcutaneous layers (Figure 10, edge areas). The damage to adjacent tissues was especially profound in the control group, whereas the compounds decreased skin tissue damage with the following efficacy: **7** < **9** < **3**. The infectious process was also accompanied by the formation of a detritus layer. In the control group at day 7, it was generally dense and closely attached to the wound surface (Figure 10a). In treated animals, it was reduced and appeared as an amorphous detachable substance. At day 14, detritus was further diminished, especially in the case of **3** (Figure 10b).

The persistent wound infection, even when inhibited by the compounds, resulted in impaired wound regeneration and contraction in all groups to different extents. The contraction rate (CR) was presented as a relative wound size, assuming the CRs of initial and fully contracted wounds to be 0 and 100%, respectively. The control wounds were weakly contracted during the observation period, in conjunction with considerable tissue damage.

The applied compounds promoted wound contraction, which is mainly driven by the subcutaneous muscle in rats [42]. This effect was enhanced at day 14 compared to day 7 (Figure 11c), indicating the role of infection inhibition in restoring contractile ability. Even in the treated groups, the remaining infection resulted in a relatively low CR. As a compensation process, surface wound shrinkage with ingrowth of skin tissues was observed, leading to a cone-shaped wound profile (Figure 10). Such a profile resembles that of poorly healing wounds, whereas repair of acute wounds typically involves increased contraction of subcutaneous muscle and de novo formation of dermis and epidermis in the lesion area [42]. **3** caused more profound surface shrinkage of wounds than the other compounds (Figure 10), along with some tendency to provide higher contraction of underlying tissues (Figure 11c).

**Figure 12:**
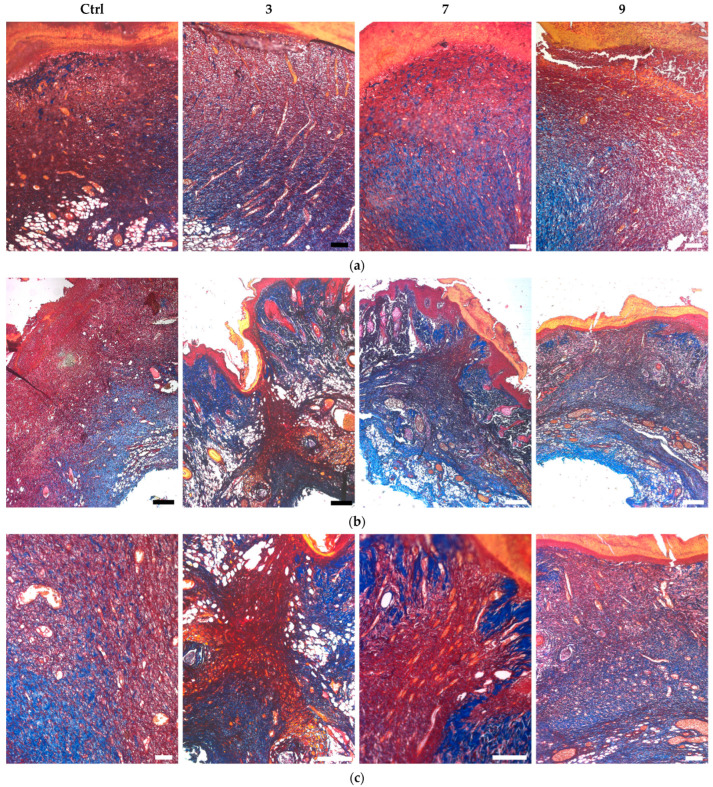
Mallory-stained sections of infected wounds treated with silver-containing compounds (**a**) at day 7 and (**b**,**c**) day 14. Scale bars are (**a**,**c**) 200 µm and (**b**) 500 µm.

Mallory trichrome staining showed that all the treated wounds at day 7 contained a higher amount of blue-stained collagen structures in newly-formed matrix (compared to the control group), although this matrix mainly consisted of non-structured fibrous tissue (Figure 12a). At day 14 it was replaced by ingrowing skin tissues with preserved dermal and appendage structures instead of de novo formation of skin layers in the lesion. In contrast to the treated groups, the control wounds at day 14 still contained unstructured fibrous matrix (Figure 12b).

In summary, the histological analysis provided consistent data supporting the ability of the applied silver-polygalaturonate complexes to inhibit persistent Staphylococcus infection in excisional wounds, thus promoting the healing process. NP complex **7** showed comparable wound healing activity to silver proteinate **9,** regardless of lower silver content of the former compound. Considering the similar characteristics of the NP component in **7** and **9**, these data may support increased in vivo antibacterial potential of the polygalacturonate ligand (Figure 5 and Figure 6). In comparison with **7**, the polygalacturonate complex with ionic silver **3** demonstrated an increased inhibitory effect on wound infection.

### 3.10. Susceptibility of Intrahospital Infections to Compounds

To further evaluate the therapeutic potential of the synthesized compounds, it was of particular importance to compare the antibacterial effect of **3**, **7**, and **9** against clinically isolated drug-resistant bacteria. For this analysis, a diffusion susceptibility test was carried out on 15 pre-characterized bacterial strains from the Republican Clinical Hospital’s collection (Table 4). All the compounds completely lysed most persistent bacteria, including *A. baumanii* (five strains), *P. aeruginosa* (two strains), MRSA-resistant *S. aureus* (three strains), and *K. pneumonia* (two strains), in accordance with the expected bactericidal activity of the silver compounds. Currently, *A. baumanii* infection is characterized by a limited number of effective medicinal and antiseptic agents. Furthermore, the compounds differently affected the growth of three other resistant *K. pneumonia* strains, including one with a mucoid phenotype. In the case of these bacteria, only complex **3** was absolutely effective, whereas compounds **7** and **9** allowed the formation of individual colonies in the inhibition zone, indicating resistance acquisition. Such specificity is presumably due to the polygalacturonate complex with ionic silver ensuring a higher effective concentration of Ag^+^, which may be critical to both eliminating resistant species and coping with tissue infections. The mechanisms underlying the increased activity of the polygalacturonate complexes with ionic silver will be additionally studied elsewhere.

## 4. Discussion

Among natural anionic polysaccharides such as glycan carboxylates (alginic acid, pectin, xanthan, gellan) and glycan sulfates (carrageenan, agaran, fucoidan), PPSs have lower MW and solution viscosity [41], which favor preparation of bioactive metallocomplexes. Most proposed silver-PPS complexes belong to NP formulations obtained upon thermal hydrolysis of pectin [21]. These formulations possess different structural, physicochemical, and biological properties in association with the variable chemistries and reducing abilities of PPS ligands [22,23]. Effective preparation of ionic SCs with pristine PPS is complicated by the low solubility of the products.

Polygalacturonic acid/polygalacturonate is a modified pectin with increased solubility and structural homogeneity, and therefore with potentially extended biomedical applications. It is obtained by enzymatic or chemical hydrolysis of PPS. As shown in [43], hydrolysates of citrus pectin obtained using pectin lyase and methyl esterase exhibited antibacterial (bactericidal) activity per se, with low dependence on the MW (1–353 kDa) and DE (11.6–60%) of the products. The mechanisms of their activity are poorly studied, also given the lack of hydrolysate purification prior analysis.

Earlier, commercially available polygalacturonic acid was used to obtain nanoprecipitated formulation by mixing sodium polygalacturonate solution with ethanol antisolvent [44]. Spontaneous reduction of Ag^+^ in the presence of excess polygalacturonate was conducted to produce ca. 10 nm-sized silver NPs capped with polygalacturonate molecules. pH-dependent association of the capping molecules was shown to affect the shape and optical properties of the resulting colloidal system [31].

To the best of our knowledge, there is a single study on the antibacterial activity of silver-polygalacturonate formulations. It reports on polygalacturonate-stabilized NPs incorporated into composite electrospun nanofiber materials [11]. The lack of quantitative data on their antibacterial effects and the use of infection-free wound model complicated comparison of these data with our results.

The main objectives of our study included (i) the development of optimized and standardized complexes of ionic and NP silver bound to polygalacturonate ligand as improved antibacterial products; (ii) characterization of the antibacterial and cytotoxic profile of these SCs depending on silver state and relative content; (iii) evaluation of the antibacterial/regenerative potential of the selected complexes in an infected wound model. In addition to the practical relevance of this work, it also provides accurate comparison of the bioeffects of ionic and NP silver formulated with the same polymer ligand, whereas the existing data for related SCs [5,6] do not reveal consistent structure-activity relationships.

In our study, sodium polygalacturonate was produced by alkaline hydrolysis of pectin, isolated in pure powder form and characterized by a series of structural analysis methods including NMR spectroscopy, which verified the product as predominantly homogalacturonan. It showed the lack of methoxy groups in contrast to the enzymatically produced polygalacturonic acid, with DE ≥ 12% [43]. Pre-oxidized and reduced polygalacturonate standards were additionally synthesized in our work to characterize the intrinsic reducing ability of the polysaccharide toward Ag^+^.

We demonstrated that under/below physiological temperature, this ability was low (<1% of total silver amount), even when an increased ligand to metal ratio was used (DS ≤ 2.5%) (Figure 2 and Figure 3). Therefore, spontaneous Ag^+^ reduction by polygalacturonate ligand should not interfere with directed NP synthesis using additional reductants, nor result in a significant amount of NP admixture in the ionic SCs. The use of NaBH_4_ reductant allowed the generation of small and quite uniform silver NP complexes with polygalacturonate in the range of metal to ligand ratio used, whereas polygalacturonate-mediated Ag^+^ reduction was accompanied by the formation of non-homogeneous nanostructures. Previously, depending on PPS structure (homogalacturonan, rhamnogalacturonan, xylogalacturonan) and reaction conditions (temperature, pH), ‘green’-synthesized silver NPs demonstrated variable sizes and shapes from spherical to nanofibrous [45,46].

The ionic and NP complexes **1**–**8** with varying silver content were synthesized with high yield and purified. The products represented water-soluble mixed polygalacturonates, in which the initial sodium amount (100%) was partially replaced by silver (DS = 1–10%). The NP component in **5**–**8** was verified spectrophotometrically and microscopically. Elemental analysis showed that silver was quantitatively incorporated into the complexes from an AgNO_3_ precursor, favoring standardization of the products.

The synthesized SCs contained almost spherical and low-dispersed NPs, with a mean size of ca. 5 nm (Figure 5 and Figure 6). Generally, bigger silver NPs were reported when PPSs were used as a reducing/capping agent [21], including low methoxylated pectin (ca. 8 nm, [47]) and polygalacturonic acid (ca. 10 nm, [31]). The mean particle size in complexes **7** and **8** was very close to that in clinically used silver proteinate **9**, which was selected as a reference agent.

Comparative analysis of the bacteriostatic and cytotoxic activities of the synthesized SCs with varying silver contents revealed important data. All the complexes were effective against both gram-positive and gram-negative bacteria, with MIC values ≥100 µg/mL, and their activity was generally determined by absolute silver amount, irrespective of the silver state (Ag^+^/Ag^0^) and polymer ligand nature (Table 2). The detected MIC values of **9** were consistent with reported ones (5.6–180 µg/mL depending on bacterial load of 10^5^–10^9^ CFU/mL [48]). Therefore, the ca. two-fold lower activity of **4** and **8** (with the highest silver content) in comparison with **9** (Table 2) is mainly due to different silver loading. To the best of our knowledge, no such comparison of antibacterial activity of polymer-formulated ionic and NP silver was made earlier, including isolation of pure SCs and their structural analysis.

Regarding particulate SCs, it is recognized that their antibacterial activity generally increases with decreasing NP size [49,50,51,52], apparently in association with size-dependent permeability and reactivity (including Ag^+^ release). Comparison of NP complexes **5**–**8** and their ionic counterparts **1**–**4** showed that these compounds were equally effective against non-resistant bacteria, as if small 5 nm NPs ensured similar availability of the silver component toward bacterial cells to that of Ag^+^-based complexes. Previously, the size-dependent antibacterial effect of laser-generated and fractioned NPs in the size range 19–47 nm was established, with additional evidences of the high activity of a smaller NP fraction (<15 nm), which however was not quantified due to poor yield of these NPs [49].

In the case of mammalian cells, the compared groups of SCs showed different activities. Although the NP complexes provided some cytotoxicity toward 3T3 and HSF cells, it was noticeably lower compared to the ionic counterparts. Moreover, extended cell culture, which could increase membrane permeability due to cell division, considerably promoted this effect on faster proliferating 3T3 cells (Table 3), indicating the role of cellular uptake of the complexes in decreasing cell viability. Consistent with this, different susceptibility to silver NPs was reported for undifferentiated and differentiated cells that should differ in their uptake profile [8]. Furthermore, the abovementioned 19–47 nm NPs were found to be non-cytotoxic (the data for the smaller NP fraction were not provided, possibly due to cytotoxic effect) [49]. Together, this indicates the existence of a critical size of silver NPs to affect cell viability.

Among the reported mechanisms of the antibacterial activity of SCs [3,5,12], targeting redox homeostasis remains poorly understood. Whereas some studies report on the antioxidant and anti-inflammatory properties of Ag^0^ NPs potentially acting as electron donors and radical scavengers [11,12], the more accepted effect of SCs involves generation of different ROS inside bacterial cells, including hydrogen peroxide, superoxide, and hydroxyl radicals [5].

Using an MCB assay for adhered cells [33], we detected an early decreasing effect of all the SCs on intracellular levels of GSH, and this was similar for the ionic and NP complexes with different silver amounts, probably due to saturating conditions (Figure 8a). The concomitant ROS overproduction in the cells was relatively low (Figure 8b), as could be expected for prooxidant compounds [53] but it showed higher dependence on silver state and content in the SCs. The detected SC-mediated aggregation of suspended bacterial cells complicated their fluorometric probing. As shown in [54], the interrelated prooxidant and antibacterial effects of 10-nm silver NPs on *E. coli* decreased as follows: Ag^+^, citrate-stabilized NPs, NPs capped with thiol-containing ligands, reflecting the role of availability/reactivity of the formulated silver component.

The in vivo antibacterial and regenerative potential of the selected SCs **3**, **7**, and **9** was studied using an *S. aureus*-infected excisional wound model. Sustained wound infection with lack of self-regenerative capacity during the 14 day observation period was verified (Figure 10, Figure 11 and Figure 12). These conditions model the chronic wound-healing process, which represents clinical cases requiring intense antibacterial therapy [55,56]. The swab and histological analyses confirmed the ability of the topically applied SCs to significantly mitigate bacterial infection and restore wound closure (Figure 9, Figure 10, Figure 11 and Figure 12). Even despite the lack of granulation tissue formation in the groups of treated animals, which could indicate normalization of the healing process, the histological data reveal high therapeutic effect of the administered complexes, considering the quite severe character of the wound infection.

Importantly, the anti-infection effect of the complexes increased from **7** ≤ **9** < **3**, demonstrating better specific activity of the polygalacturonate complex with ionic silver in vivo. Furthermore, given the different silver content in the NP-based compounds **7** and **9** (ca. 2.5% vs. 7.6%), the polygalacturonate ligand seems to better contribute to the activity of formulated silver than the polypeptide ligand under these conditions. Additional testing of the compared compounds on clinically isolated drug-resistant bacteria also revealed a better antibacterial profile of **3** compared to **7** and **9** (Table 4). Based on these results, the Ag^+^-based polygalacturonate complex can be considered as a more potent agent for treatment of bacterial infections compared to the NP-based counterparts. Taking together the in vitro and in vivo data, it can be supposed that the bioactivity of the ionic complexes **1**–**4** increases relative to the **5**–**8** counterparts as the living test system becomes more complex (i.e., bacteria, mammalian cells, skin tissues), encouraging further study of the mechanism of action of the compounds.

## 5. Conclusions

This study proposes chemically modified polygalacturonate as a polymer ligand for antibacterial silver-containing complexes with defined structure. Pre-synthesized sodium polygalacturonate was reacted with AgNO_3_ to obtain sodium/silver polygalacturonates with controllable stoichiometry. By treating with NaBH_4_, these polygalacturonates were effectively converted into homogenous silver nanoparticle counterparts with a small NP size of ca. 5 nm. Pre-oxidized and pre-reduced polygalacturonate standards were obtained to verify the intrinsic reducing ability of sodium polygalacturonate, and its contribution to the formation of particulate admixtures was assessed as a part of standardization of the complexes.

The following structure-activity relationships of the complexes were revealed. When targeting bacteria, their activity is mainly determined by absolute silver amount rather than complex composition, silver state, and ligand specificity (i.e., polysaccharide vs. protein), as if the small nanoparticle component affected bacterial targets in a similar manner to the ionic counterpart. The potential exception is multidrug-resistant bacteria, as shown for some *K. pneumonia* strains fully susceptible only to the Ag^+^-polygalacturonate complex. In the case of mammalian cells, in addition to silver amount, other factors contributed to cytotoxicity of the compounds including cell type, culture duration, and silver state. All the complexes caused depletion of intracellular levels of reduced glutathione and some overproduction of ROS, suggesting oxidative stress as a probable mechanism underlying the cytotoxicity of the compounds.

Our in vivo study demonstrated the comparable potential of topically applied polygalacturonate complexes containing ionic and nanoparticle silver, as well as silver proteinate with similar nanoparticle characteristics, in mitigating bacteria and promoting healing in persistently infected wounds. Nevertheless, our data support that the polygalacturonate complexes, especially ionic ones, can provide better outcomes than clinically used silver proteinate, encouraging further preclinical investigation of the synthesized compounds.

## Figures and Tables

**Table 1:** Experimental conditions for the preparation of polygalacturonate complexes with silver ions and nanoparticles (40 °C) and their characteristics.

No	AgNO_3_ *	NaBH_4_ *	Na^+^:Ag^+^ Mol. Ratio, %	Characteristics
**1**	7.76	–	99:1	Yield 89%. Moisture 5.7%. FTIR (ν/cm^−1^): 3436, 2937, 1616, 1415, 1332, 1238, 1146, 1101, 1016, 950, 891, 835, 755, 637. Metal content (%): Na 11.08; Ag 0.49
**2**	19.92	–	97.5:2.5	Yield 93%. Moisture 6.4%. FTIR (ν/cm^−1^): 3417, 2932, 1611, 1422, 1332, 1238, 1101, 1016, 951, 891, 836, 766, 642. Metal content (%): Na 10.80; Ag 1.32
**3**	38.76	–	95:5	Yield 90%. Moisture 5.4%. FTIR (ν/cm^−1^): 3432, 2934, 1610, 1421, 1330, 1237, 1148, 1101, 1013, 951, 893, 835, 764, 638. Metal content (%): Na 10.53; Ag 2.63
**4**	77.52	–	90:10	Yield 91%. Moisture 6.1%. FTIR (ν/cm^−1^): 3437, 2931, 1614, 1414, 1331, 1239, 1146, 1102, 1016, 949, 894, 835, 639. Metal content (%): Na 10.00; Ag 5.18
**5**	7.76	0.9	99:1	Yield 90%. Moisture 5.4%. FTIR (ν/cm^−1^): 3232, 2934, 1609, 1415, 1332, 1237, 1148, 1098, 1013, 950, 892, 835, 766, 638. Metal content (%): Na 11.15; Ag 0.5
**6**	19.92	2.2	97.5:2.5	Yield 93%. Moisture 6.4%. FTIR (ν/cm^−1^): 3435, 2930, 1611, 1414, 1331, 1238, 1149, 1100, 1015, 951, 892, 835, 764, 638. Metal content (%): Na 11.08; Ag 1.26
**7**	38.76	4.4	95:5	Yield 91%. Moisture 5.3%. FTIR (ν/cm^−1^): 3430, 2928, 1619, 1414, 1330, 1237, 1149, 1100, 1015, 950, 893, 835, 770, 635. Metal content (%): Na 11.08; Ag 2.48.
**8**	77.52	8.8	90:10	Yield 92%. Moisture 6.8%. FTIR (ν/cm^−1^): 3430, 2930, 1608, 1413, 1331, 1234, 1146, 1098, 1012, 950, 891, 834, 764, 639. Metal content (%): Na 11.19; Ag 5.06

* Relative contents of AgNO_3_ or NaBH_4_ in mg per g of polygalacturonate (absolute dry weight).

**Table 2:** MIC values of silver-polygalacturonate complexes (mg/mL, the left uncolored panel) and corresponding concentrations of elemental silver (µg/mL, the right grey-colored panel).

No	*E.c.*	*K.p.*	*S.a.*	*B.s.*	*E.c.*	*K.p.*	*S.a.*	*B.s.*
**1**	2.4	1.2	2.4	2.4	8.8	4.4	8.8	8.8
**2**	1.2	0.6	1.2	0.6	11	5.4	11	5.4
**3**	1.2	0.3	0.6	0.3	21.6	5.4	10.8	5.4
**4**	0.3	0.1	0.6	0.3	10.4	5.3	20.9	10.5
**5**	2.3	1.2	2.3	2.3	8.6	4.3	8.6	8.6
**6**	1.2	0.6	1.2	0.6	10.7	5.4	10.7	5.4
**7**	0.3	0.3	0.6	0.3	5.1	5.2	10.4	5.2
**8**	0.2	0.2	0.3	0.2	5.2	5.2	10.4	5.2
**9**	0.1	0.1	0.2	0.1	5.7	5.7	11.4	5.7

*E.c.—Escherichia coli*; *K.p.—Klebsiella pneumonia*; *S.a.—Staphylococcus aureus*; *B.s.—Bacillus subtilis*.

**Table 3:** IC_50_ values of silver-containing compounds (µg/mL) according to resazurin assay after 24 h (the left uncolored panels) and 72 h (the right grey-colored panels) of exposure of 3T3 cells and HSF.

No	3T3 Cells		HSF	
**1**	>2000	210.2 ± 20	708.7 ± 44.3	557.1 ± 71
**2**	1100 ± 30	55.3 ± 40	323.2 ± 49.6	288.6 ± 35.0
**3**	760 ± 40	33.7 ± 10	228.4 ± 43.7	124.8 ± 101.4
**4**	500 ± 100	25 ± 10	185.4 ± 77.1	155.1 ± 64
**5**	>2000	403 ± 19	1280 ± 45.8	1436.8 ± 31.2
**6**	>2000	327.3 ± 46	1262 ± 12.4	605.8 ± 95.6
**7**	>2000	107.6 ± 56.7	1141 ± 84.8	486.0 ± 61
**8**	>2000	76.6 ± 48.4	598.1 ± 50.6	418.3 ± 195.4
**9**	1200 ± 210	46.7 ± 20	193.2 ± 29.1	199.5 ± 34.6

**Table 4:** Susceptibility profile of hospital-derived drug-resistant bacteria to silver-containing compounds (S—sensitive, R—resistant).

Bacteria	Antibiotic Resistance	Susceptibility
*Acinetobacter baumanii* (strains 2091, 2092, 2097, 2102, 2110)	amikacin, levofloxacin, imipenem, meropenem, trimethoprim/sulfamethoxazole	**3**—S **7**—S **9**—S
*Pseudomonas aeruginosa* (strains 2095, 2175)	ceftazidime, imipenem, meropenem, levofloxacin, amikacin	**3**—S **7**—S **9**—S
*Staphylococcus aureus* (MRSA-resistant strains 2103, 2126, 2174)	methicillin, cefoxitin, erythromycin, clindamycin, levofloxacin	**3**—S **7**—S **9**—S
*Klebsiella pneumonia* (strains 2120, 2173)	amoxiclav, ceftazidime, cefepime, meropenem, amikacin, levofloxacin	**3**—S **7**—S **9**—S
*Klebsiella pneumonia* (strains 2094, 2149)	amoxiclav, ceftazidime, cefepime, meropenem, amikacin, levofloxacin	**3**—S **7**—R **9**—R
*Klebsiella pneumonia* (mucoid strain 2128)	amoxiclav, ceftazidime, cefepime, meropenem, amikacin, levofloxacin	**3**—S **7**—R **9**—R

## Data Availability

The original contributions presented in this study are included in the article/Supplementary Material, and further inquiries can be directed to the corresponding author.

## Supplementary Materials

The following supporting information can be downloaded at: https://www.mdpi.com/article/10.3390/polym17202798/s1, Figure S1: Concentration dependences of the electroconductivity of pectin (**a**) and sodium polygalacturonate (**b**) in aqueous solution; Figure S2: Changes in the (**a**,**b**) hydrodynamic diameter and (**c**,**d**) particle dispersion index of silver-containing complexes upon storage in aqueous solution (1 mg/mL); Figure S3: Representative TEM images of silver-polygalacturonate complex **3** (upper panel) and complex **4** (lower panel) at different magnifications; Figure S4: Concentration-dependent cytotoxicity of silver-containing complexes toward (**a**) 3T3 cells and (**b**) HSFs (resazurin assay, 72 h); Figure S5: Effect of silver-containing complexes on ROS generation in 3T3 cells according to DCFDA fluorescence; Figure S6: IR spectrum of sodium polygalacturonate; Figure S7: IR spectrum of oxidized sodium polygalacturonate (PGNa_Ox); Figure S8: IR spectrum of reduced sodium polygalacturonate (PGNa_Red); Figure S9: IR spectrum of silver polygalacturonate **1**; Figure S10: IR spectrum of silver polygalacturonate **2**; Figure S11 IR spectrum of silver polygalacturonate **3**; Figure S12: IR spectrum of silver polygalacturonate **4**; Figure S13: IR spectrum of silver polygalacturonate **5**; Figure S14: IR spectrum of silver polygalacturonate **6**; Figure S15: IR spectrum of silver polygalacturonate **7**; Figure S16: IR spectrum of silver polygalacturonate **8**; Figure S17: IR spectrum of silver-polygalacturonate complex prepared at RT (Na:Ag ratio 99:1); Figure S18: IR spectrum of silver-polygalacturonate complex prepared at RT (Na:Ag ratio 19:1); Figure S19: IR spectrum of silver-polygalacturonate complex prepared at RT (Na:Ag ratio 9:1); Figure S20: IR spectrum of silver-polygalacturonate complex prepared at 60 °C (Na:Ag ratio 99:1); Figure S21: IR spectrum of silver-polygalacturonate complex prepared at 60 °C (Na:Ag ratio 19:1); Figure S22: IR spectrum of silver-polygalacturonate complex prepared at 60 °C (Na:Ag ratio 9:1); Table S1: Characteristics of silver-polygalacturonate complexes prepared at RT (left panel) and 60 °C (right panel); Table S2: MIC values (mg/mL) of silver-containing complexes: as-prepared (left panels) and after storage for 7 days (right panels).

## References

[1] Gomes H.I.O., Martins C.S.M., Prior J.A.V. (2021). Silver Nanoparticles as Carriers of Anticancer Drugs for Efficient Target Treatment of Cancer Cells. Nanomaterials.

[2] Godoy-Gallardo M., Eckhard U., Delgado L.M., de Roo Puente Y.J.D., Hoyos-Nogues M., Gil F.J., Perez R.A. (2021). Antibacterial approaches in tissue engineering using metal ions and nanoparticles: From mechanisms to applications. Bioact. Mater..

[3] Medici S., Peana M., Nurchi V.M., Zoroddu M.A. (2019). Medical Uses of Silver: History, Myths, and Scientific Evidence. J. Med. Chem..

[4] Mijnendonckx K., Leys N., Mahillon J., Silver S., Van Houdt R. (2013). Antimicrobial silver: Uses, toxicity and potential for resistance. Biometals.

[5] Kedziora A., Speruda M., Krzyzewska E., Rybka J., Lukowiak A., Bugla-Ploskonska G. (2018). Similarities and Differences Between Silver Ions and Silver in Nanoforms as Antibacterial Agents. Int. J. Mol. Sci..

[6] Hadrup N., Sharma A.K., Loeschner K. (2018). Toxicity of silver ions, metallic silver, and silver nanoparticle materials after in vivo dermal and mucosal surface exposure: A review. Regul. Toxicol. Pharmacol..

[7] Bajwa N.A., Mazurek K., Chebolu E., Pourafkari L. (2021). Argyria: A cause of pseudocyanosis. QJM.

[8] Noga M., Milan J., Frydrych A., Jurowski K. (2023). Toxicological Aspects, Safety Assessment, and Green Toxicology of Silver Nanoparticles (AgNPs)-Critical Review: State of the Art. Int. J. Mol. Sci..

[9] Zhao Z.Y., Li P.J., Xie R.S., Cao X.Y., Su D.L., Shan Y. (2022). Biosynthesis of silver nanoparticle composites based on hesperidin and pectin and their synergistic antibacterial mechanism. Int. J. Biol. Macromol..

[10] Li S., Wei N., Wei J., Fang C., Feng T., Liu F., Liu X., Wu B. (2024). Curcumin and silver nanoparticles loaded antibacterial multifunctional pectin/gelatin films for food packaging applications. Int. J. Biol. Macromol..

[11] El-Aassar M.R., Ibrahim O.M., Fouda M.M.G., El-Beheri N.G., Agwa M.M. (2020). Wound healing of nanofiber comprising Polygalacturonic/Hyaluronic acid embedded silver nanoparticles: In-vitro and in-vivo studies. Carbohydr. Polym..

[12] Rybka M., Mazurek L., Konop M. (2022). Beneficial Effect of Wound Dressings Containing Silver and Silver Nanoparticles in Wound Healing—From Experimental Studies to Clinical Practice. Life.

[13] Sood K., Shanavas A. (2024). Autologous serum protein stabilized silver quantum clusters as host-specific antibacterial agents. Nanomedicine.

[14] Duran N., Silveira C.P., Duran M., Martinez D.S. (2015). Silver nanoparticle protein corona and toxicity: A mini-review. J. Nanobiotechnol..

[15] Lai W., Wang Q., Li L., Hu Z., Chen J., Fang Q. (2017). Interaction of gold and silver nanoparticles with human plasma: Analysis of protein corona reveals specific binding patterns. Colloids Surf. B Biointerfaces.

[16] Batista C.C.S., Panico K., Trousil J., Janouskova O., de Castro C.E., Stepanek P., Giacomelli F.C. (2022). Protein coronas coating polymer-stabilized silver nanocolloids attenuate cytotoxicity with minor effects on antimicrobial performance. Colloids Surf. B Biointerfaces.

[17] Zaitseva O., Khudyakov A., Sergushkina M., Solomina O., Polezhaeva T. (2020). Pectins as a universal medicine. Fitoterapia.

[18] Ciriminna R., Fidalgo A., Meneguzzo F., Presentato A., Scurria A., Nuzzo D., Alduina R., Ilharco L.M., Pagliaro M. (2020). Pectin: A Long-Neglected Broad-Spectrum Antibacterial. ChemMedChem.

[19] Wang R., Liang R., Dai T., Chen J., Shuai X., Liu C. (2019). Pectin-based adsorbents for heavy metal ions: A review. Trends Food Sci. Technol..

[20] Kartel M.T., Kupchik L.A., Veisov B.K. (1999). Evaluation of pectin binding of heavy metal ions in aqueous solutions. Chemosphere.

[21] Devasvaran K., Lim V. (2021). Green synthesis of metallic nanoparticles using pectin as a reducing agent: A systematic review of the biological activities. Pharm. Biol..

[22] Roman-Benn A., Contador C.A., Li M.-W., Lam H.-M., Ah-Hen K., Ulloa P.E., Ravanal M.C. (2023). Pectin: An overview of sources, extraction and applications in food products, biomedical, pharmaceutical and environmental issues. Food Chem. Adv..

[23] Chandel V., Biswas D., Roy S., Vaidya D., Verma A., Gupta A. (2022). Current Advancements in Pectin: Extraction, Properties and Multifunctional Applications. Foods.

[24] Minzanova S.T., Mironov V.F., Vyshtakalyuk A.B., Tsepaeva O.V., Mironova L.G., Mindubaev A.Z., Nizameev I.R., Kholin K.V., Milyukov V.A. (2015). Complexation of pectin with macro- and microelements. Antianemic activity of Na, Fe and Na, Ca, Fe complexes. Carbohydr. Polym..

[25] Minzanova S., Mironov V., Vyshtakalyuk A., Tsepaeva O., Mindubaev A., Mironova L., Zobov V., Lenina O., Lantsova A., Konovalov A. (2009). Scientific grounds and process aspects for the production of polygalacturonate with Ca^2+^ and Fe^2+^ ions. Dokl. Chem..

[26] Minzanova S., Khamatgalimov A., Ryzhkina I., Murtazina L., Mironova L., Kadirov M., Vyshtakalyuk A., Milyukov V., Mironov V. (2016). Synthesis and physicochemical properties of antianemic iron and calcium complexes with sodium polygalacturonate. Dokl. Phys. Chem..

[27] Minzanova S., Mironov V., Khabibullina A., Arkhipova D., Mironova L., Nemtarev A., Vyshtakalyuk A., Chekunkov E., Kholin K., Nizameev I. (2021). New metal complexes of citrus pectin with magnesium ions: Synthesis, properties, and immunomodulatory activity. Russ. Chem. Bull..

[28] Minzanova S., Mironov V., Mironova L., Nemtarev A., Vyshtakalyuk A., Kholin K., Nizameeva G., Milyukov V. (2019). Synthesis, properties, and antianemic activity of new metal complexes of sodium pectinate with iron and calcium. Russ. Chem. Bull..

[29] Nizameev I.R., Kadirov D.M., Nizameeva G.R., Sabirova A.F., Kholin K.V., Morozov M.V., Mironova L.G., Zairov R.R., Minzanova S.T., Sinyashin O.G. (2022). Complexes of Sodium Pectate with Nickel for Hydrogen Oxidation and Oxygen Reduction in Proton-Exchange Membrane Fuel Cells. Int. J. Mol. Sci..

[30] Kholin K.V., Sabirova A.F., Kadirov D.M., Khamatgalimov A.R., Khrizanforov M.N., Nizameev I.R., Morozov M.V., Gainullin R.R., Sultanov T.P., Minzanova S.T. (2023). Carbonized Nickel Complex of Sodium Pectate as Catalyst for Proton-Exchange Membrane Fuel Cells. Membranes.

[31] Gasilova E.R., Alexandrova G.P., Tyshkunova I.V., Dubashynskaya N.V., Vlasova E.N., Romanov D.P. (2023). Optically active pH-dependent colloids of silver nanoparticles capped by polygalacturonic acid. J. Nanopart. Res..

[32] (2012). Methods for Dilution Antimicrobial Susceptibility Tests for Bacteria That Grow Aerobically, 9th ed..

[33] Ishkaeva R.A., Zoughaib M., Laikov A.V., Angelova P.R., Abdullin T.I. (2022). Probing Cell Redox State and Glutathione-Modulating Factors Using a Monochlorobimane-Based Microplate Assay. Antioxidants.

[34] Perni S., Alotaibi H.F., Yergeshov A.A., Dang T., Abdullin T.I., Prokopovich P. (2020). Long acting anti-infection constructs on titanium. J. Control. Release.

[35] Yergeshov A.A., Zoughaib M., Ishkaeva R.A., Savina I.N., Abdullin T.I. (2022). Regenerative Activities of ROS-Modulating Trace Metals in Subcutaneously Implanted Biodegradable Cryogel. Gels.

[36] Yang L., Yergeshov A.A., Al-Thaher Y., Avdokushina S., Statsenko E., Abdullin T.I., Prokopovich P. (2023). Nanocomposite orthopaedic bone cement combining long-acting dual antimicrobial drugs. Biomater. Adv..

[37] Harding S.E., Berth G., Ball A., Mitchell J.R., de la Torre J.G. (1991). The molecular weight distribution and conformation of citrus pectins in solution studied by hydrodynamics. Carbohydr. Polym..

[38] Tewari Y.B., Goldberg R.N. (1991). Thermodynamics of hydrolysis of disaccharides: Lactulose, α-d-melibiose, palatinose, d-trehalose, d-turanose and 3-o-β-d-galactopyranosyl-d-arabinose. Biophys. Chem..

[39] Knirel Y.A., Naumenko O.I., Sof’ya N.S., Perepelov A.V. (2019). Chemical methods for selective cleavage of glycosidic bonds in the structural analysis of bacterial polysaccharides. Russ. Chem. Rev..

[40] Paramelle D., Sadovoy A., Gorelik S., Free P., Hobley J., Fernig D.G. (2014). A rapid method to estimate the concentration of citrate capped silver nanoparticles from UV-visible light spectra. Analyst.

[41] Barras F., Aussel L., Ezraty B. (2018). Silver and Antibiotic, New Facts to an Old Story. Antibiotics.

[42] Gross J., Farinelli W., Sadow P., Anderson R., Bruns R. (1995). On the mechanism of skin wound” contraction”: A granulation tissue” knockout” with a normal phenotype. Proc. Natl. Acad. Sci. USA.

[43] Wu M.C., Li H.c., Wu P.H., Huang P.H., Wang Y.T. (2014). Assessment of oligogalacturonide from citrus pectin as a potential antibacterial agent against foodborne pathogens. J. Food Sci..

[44] Gasilova E.R., Aleksandrova G.P., Tyshkunova I.V. (2022). Colloidal nanoparticles of sodium polygalacturonate prepared by nanoprecipitation. Carbohydr. Polym..

[45] Zhang W., Zhao X.J., Jiang Y., Zhou Z. (2017). Citrus pectin derived silver nanoparticles and their antibacterial activity. Inorg. Nano-Met. Chem..

[46] Li K., Cui S., Hu J., Zhou Y., Liu Y. (2018). Crosslinked pectin nanofibers with well-dispersed Ag nanoparticles: Preparation and characterization. Carbohydr. Polym..

[47] Pallavicini P., Arciola C.R., Bertoglio F., Curtosi S., Dacarro G., D’Agostino A., Ferrari F., Merli D., Milanese C., Rossi S. (2017). Silver nanoparticles synthesized and coated with pectin: An ideal compromise for anti-bacterial and anti-biofilm action combined with wound-healing properties. J. Colloid. Interface Sci..

[48] Rzheuzsky S.E., Dovnar A.G., Kugach V.V. (2015). Study of antimicrobial activity of the funiargol. Bull. Vitebsk. State Med. Univ..

[49] Korshed P., Li L., Liu Z., Mironov A., Wang T. (2019). Size-dependent antibacterial activity for laser-generated silver nanoparticles. J. Interdiscip. Nanomed..

[50] Demchenko V., Riabov S., Sinelnikov S., Radchenko O., Kobylinskyi S., Rybalchenko N. (2020). Novel approach to synthesis of silver nanoparticles in interpolyelectrolyte complexes based on pectin, chitosan, starch and their derivatives. Carbohydr. Polym..

[51] Yang E.J., Kim S., Kim J.S., Choi I.H. (2012). Inflammasome formation and IL-1beta release by human blood monocytes in response to silver nanoparticles. Biomaterials.

[52] Guo X., Li Y., Yan J., Ingle T., Jones M.Y., Mei N., Boudreau M.D., Cunningham C.K., Abbas M., Paredes A.M. (2016). Size- and coating-dependent cytotoxicity and genotoxicity of silver nanoparticles evaluated using in vitro standard assays. Nanotoxicology.

[53] Tsepaeva O.V., Salikhova T.I., Grigor’eva L.R., Ponomaryov D.V., Dang T., Ishkaeva R.A., Abdullin T.I., Nemtarev A.V., Mironov V.F. (2021). Synthesis and in vitro evaluation of triphenylphosphonium derivatives of acetylsalicylic and salicylic acids: Structure-dependent interactions with cancer cells, bacteria, and mitochondria. Med. Chem. Res..

[54] Long Y.M., Hu L.G., Yan X.T., Zhao X.C., Zhou Q.F., Cai Y., Jiang G.B. (2017). Surface ligand controls silver ion release of nanosilver and its antibacterial activity against *Escherichia coli*. Int. J. Nanomed..

[55] Menke N.B., Ward K.R., Witten T.M., Bonchev D.G., Diegelmann R.F. (2007). Impaired wound healing. Clin. Dermatol..

[56] Bassetti M., Vena A., Castaldo N., Maruccia M., Papa G., Ricci E., Giudice G. (2023). Classification of Wound Infections. Pearls and Pitfalls in Skin Ulcer Management.

