# Supplementary material for: Synthesis and Comparative Study of the Structure and Antibacterial Activity of Polygalacturonate Complexes with Ionic and Nanoparticulate Silver
Source: Polymers (Basel). 2025 Oct 20;17(20):2798. doi: 10.3390/polym17202798 (PMC12567555; doi:10.3390/polym17202798)
Supplement: Supplementary file 1 [file polymers-17-02798-s001.zip › polymers-3917470-supplementary.pdf]

Supplementary materials

Synthesis and comparative study of the structure and antibacterial activity of polygalacturonate complexes containing ionic and nanoparticulate silver

Andrey V. Nemtarev [1,4,*], Elena V. Kuznetsova [2,3], Abdulla A. Yergeshov [2,3], Darya S. Eflova [2,3], Rezeda A. Ishkaeva [2,3], Inna R. Valiullina [5], Vladimir F. Mironov [1,4], Diana V. Salakhieva [2,3], and Timur I. Abdullin [2,3,*]

[1] Alexander Butlerov Institute of Chemistry, Kazan (Volga Region) Federal University, 420008 Kazan, Russia; avnemtarev@kpfu.ru (A.V.N.), vladimir.mironov@kpfu.ru (V.F.M.);
[2] Institute of Fundamental Medicine and Biology, Kazan (Volga Region) Federal University, 18 Kremlyovskaya St., 420008 Kazan, Russia; elenvkuznecova@kpfu.ru (E.V.K.); abdulla.ergeshov@mail.ru (A.A.E.); daseflova@kpfu.ru (D.S.E.); rezaahmadishina@kpfu.ru (R.A.I.); divsalahieva@kpfu.ru (D.V.S.);
[3] Scientific and Educational Center of Pharmaceutics, Kazan (Volga Region) Federal University, 18 Kremlyovskaya St., 420008 Kazan, Russia; elenvkuznecova@kpfu.ru (E.V.K.); abdulla.ergeshov@mail.ru (A.A.E.); daseflova@kpfu.ru (D.S.E.); rezaahmadishina@kpfu.ru (R.A.I.); divsalahieva@kpfu.ru (D.V.S.);
[4] Arbuzov Institute of Organic and Physical Chemistry, 8 Arbuzov St., 420088 Kazan, Russia; avnemtarev@kpfu.ru (A.V.N.), vladimir.mironov@kpfu.ru (V.F.M.);
[5] State Autonomous Healthcare Institution Republican Clinical Hospital of the Ministry of Health of the Republic of Tatarstan, 138 Orenburg Highway, 420064 Kazan, Russia; innalife@yandex.ru (I.R.V.)
* Correspondence: avnemtarev@kpfu.ru (A.V.N.); tabdulli@gmail.com, timur.abdullin@kpfu.ru (T.I.A.); Tel.: +7-(843)-236-7640

Table of contents

Table of contents	S1
Experimental section	S3
**Table S1.** Characteristics of silver-polygalacturonate complexes prepared at RT (left panel) and 60 °C (right panel).	S5
**Figure S1.** Concentration dependences of the electroconductivity of pectin (**a**) and sodium polygalacturonate (**b**) in aqueous solution.	S5
**Figure S2.** Changes in (**a**, **b**) hydrodynamic diameter and (**c**, **d**) particle dispersion index of silver-containing complexes upon storage in aqueous solution (1 mg/mL).	S6
**Figure S3.** Representative TEM images of silver-polygalacturonate complex **3** (upper panel) and complex **4** (lower panel) at different magnifications.	S7
**Table S2.** MIC values (mg/mL) of silver-containing complexes: as-prepared (left panels) and after storage for 7 days (right panels).	S7
**Figure S4.** Concentration-dependent cytotoxicity of silver-containing complexes for (**a**) 3T3 cells and (**b**) HSF (resazurin assay, 72 h).	S8
**Figure S5.** Effect of silver-containing complexes on ROS generation in 3T3 cells according to DCFDA fluorescence.	S8
**Figure S6.** IR spectrum of sodium polygalacturonate.	S9
**Figure S7.** IR spectrum of oxidized sodium polygalacturonate (PGNa_Ox).	S9
**Figure S8.** IR spectrum of reduced sodium polygalacturonate (PGNa_Red).	S9
**Figure S9.** IR spectrum of silver polygalacturonate **1**.	S10
**Figure S10.** IR spectrum of silver polygalacturonate **2**.	S10
**Figure S11.** IR spectrum of silver polygalacturonate **3**.	S10
**Figure S12.** IR spectrum of silver polygalacturonate **4**.	S11
**Figure S13.** IR spectrum of silver polygalacturonate **5**.	S11
**Figure S14.** IR spectrum of silver polygalacturonate **6**.	S11

**Figure S15.** IR spectrum of silver polygalacturonate **7**.	S12
**Figure S16.** IR spectrum of silver polygalacturonate **8**.	S12
**Figure S17.** IR spectrum of silver-polygalacturonate complex prepared at RT (Na:Ag ratio 99:1).	S12
**Figure S18.** IR spectrum of silver-polygalacturonate complex prepared at RT (Na:Ag ratio 19:1).	S13
**Figure S19.** IR spectrum of silver-polygalacturonate complex prepared at RT (Na:Ag ratio 9:1).	S13
**Figure S20.** IR spectrum of silver-polygalacturonate complex prepared at 60 °C (Na:Ag ratio 99:1).	S13
**Figure S21.** IR spectrum of silver-polygalacturonate complex prepared at 60 °C (Na:Ag ratio 19:1).	S14
**Figure S22.** IR spectrum of silver-polygalacturonate complex prepared at 60 °C (Na:Ag ratio 9:1).	S14

Experimental section

Materials

Phenolphthalein, carbazole, and ammonium molybdate were purchased from Acros Organics. $Na_2HAsO_4 \times 7H_2O$ was purchased from LLC 'Uralskii zavod chimreactivov' (Verkhnyaya Pyshma, Russia). Standard titers 0.1N NaOH and 0.1N HCl were purchased from LLC 'Uralkhiminvest' (Ufa, Russia). Sulfuric acid, sodium tetraborate, urea, $CuSO_4 \times 10H_2O$, cationite KU-2-8 (H^+-form), and NaF were purchased from JSC 'Vekton' (Saint Petersburg, Russia).

Methods

Determination of degree of esterification

First, 0.2 g of pectin was placed in an Erlenmeyer flask and moistened with a small amount of 95% ethanol to prevent clumping. Then, the sample was mixed with 20 mL of pre-warmed distilled water (40 °C) and stirred for 2 h until complete dissolution. The resulting solution was titrated with 0.1N NaOH (V_1) solution with a phenolphthalein indicator until becoming slightly pink. Then, 10 mL of 0.1 N NaOH solution was added, and the mixture was kept for 2 h at room temperature to saponify the esterified carboxyl groups of pectin. Finally, 10 mL of 0.1 N HCl was added to the solution, and its excess was titrated with 0.1 N NaOH (V_2) solution. The degree of esterification (DE) of pectin was calculated by the formula $DE = (V_2 / (V_1 + V_2)) \times 100\%$.

Quantification of uronide content

Calibration graph for D-galacturonic acid

First, 0.5 mL aliquots of D-galacturonic acid (D-GalA) in a concentration range of 10 to 100 μg/mL in an ice bath were gently mixed with 3 mL of reagent C. To prepare reagent C, 250 mg of $Na_2B_4O_7 \times 10H_2O$ was added to 100 mL of conc. sulfuric acid and heated until release of sulfuric anhydride, then cooled and mixed with 150 mg of urea. The resulting mixtures were thoroughly mixed and heated in a boiling water bath for 6 min. Then, they were transferred to the ice bath, and 0.1 mL of 0.2% carbazole alcohol solution was added. After thorough mixing and keeping in the boiling water bath for 10 min, the mixtures were cooled at room temperature for 40 min. The optical absorption of the resulting solutions at λ = 535 nm was determined in 1 cm quartz cuvettes against a D-galacturonic acid-free counterpart (0.5 mL of distilled water plus 3 mL of reagent C) used as a blank solution. After subtraction of the optical absorption of the carbazole-free counterpart, a calibration graph of analyte concentration vs. registered signal was constructed. The method of least squares was used for approximation. The linear approximation equation was used to determine the uronide content in pectin polysaccharides.

Uronide content in pectin and polygalacturonate

Pectin was deesterifed prior to analysis. Briefly, 75 μL of pectin solution (5 mg/mL) was placed in a 5 mL measuring flask and mixed with 75 μL of a 0.1 N NaOH solution. After keeping for 2 h at room temperature, 75 μL of a 0.1 N HCl solution was added, and the volume was adjusted to 5 mL with distilled water. The prepared test solution was used to determine the uronide content as described for D-galacturonic acid.

The uronide content in polygalacturonate was determined analogously but without pre-deesterification. The test solution contained 75 μg/mL of sodium polygalacturonate. The uronide content was calculated in terms of galacturonic anhydride (M = 176) and anhydrogalacturonic acid (M = 198) for pectin and polygalacturonate, respectively.

Determination of the molecular weight of polygalacturonate

The molecular weight of sodium polygalacturonate was determined by the end group colorimetric method using arsenomolybdate reagent. It is based on the ability of polygalacturonate to reduce copper(II) to copper(I) due to the presence of reducing terminal fragments. Copper(I) ions interact with arsenomolybdate reagent to form colored molybdenum blue in proportion to the reducing capacity. The analysis conditions, including protocols for preparation of copper reagent A and arsenomolybdate reagent B, are detailed in [doi: 0.1016/S0008-6215(00)80191-3].

Calibration graph for D-GalA

First, 1.5 mL of reagent A was added to test tubes containing 0.5 mL of standard D-GalA solutions in a concentration range of 5 to 100 μg/mL, thoroughly mixed, and heated in boiling water bath for 10 min. The samples were cooled in an ice bath without shaking, and 1 mL of reagent B was added with gentle shaking followed by the addition of 2 mL of water. After keeping for 30 min, the optical absorption was determined at λ = 600 nm relative to the blank solution containing reagent B.

Sodium polygalacturonate solutions at concentrations of ≤5 mg/mL were prepared to prevent precipitation of copper polygalacturates. Molecular weight was calculated in terms of sodium salt of anhydrogalacturonic acid (M = 198).

Purification of pectin

Commercial citrus pectin (Herbstreith and Fox) was used. To remove unbound neutral sugars, pectin was double-precipitated from 2% aqueous solution with ethanol. To remove ash (metals), a 1% aqueous solution of pectin was passed through a column with KU-2-8 cationite (full static exchange capacity is 1.8 mmol/cm^3) in H^+ form at a temperature of 10°C and a rate of 10 mL/min. The purified pectin solution was concentrated under vacuum at 50°C and precipitated with ethanol. The precipitate was dried by keeping in the open air and then under vacuum (p = 0.05 mbar) at a temperature of 40°C for 24 h. The product was ground to a particle size of <125 microns to obtain a light beige amorphous substance. Moisture 7.4%. Uronide content 73 ± 1%. DE 60 ± 0.7%. Molecular weight 27.9 ± 0.5 kDa. Kinematic viscosity 3.1507 mm^2/s (20 °C, *c* 0.5, H_2O). $[\alpha]^{20}_{D}$ + 212.6 (*c* 0.5, H_2O). IR, ν, cm^{-1}: 3431, 2936, 1748, 1635, 1443, 1370, 1231, 1148, 1105, 1051, 1019, 969, 921, 631, 590, 533. ^{13}C-{^1H} NMR (D_2O, δ_C ppm, *J* Hz): 173.08 and 170.77 (C^6, D-Gal*p*A), 104.41 (C^1, D-Gal*p*), 100.24 and 99.71 (C^1, D-Gal*p*A), 79.03, 78.62 and 78.25 (C^4, D-Gal*p*A), 77.71 (C^4, D-Gal*p*), 74.56 (C^5, D-Gal*p*), 73.37 (C^3, D-Gal*p*), 71.89 (C^2, D-Gal*p*), 70.61 (C^3, D-Gal*p*A), 68.29 and 67.99 (C^2, C^5 D-Gal*p*A), 60.66 (C^6, D-Gal*p*), 52.92 ($C(O)OCH_3$, D-Gal*p*A), 16.54 (C^6, L-Rha*p*).

Table S1. Characteristics of silver-polygalacturonate complexes prepared at RT (left panel) and 60 °C (right panel).

Na:Ag ratio	Characteristics	Na:Ag ratio	Characteristics
99:1	Yield 89%. Moisture 9.7%. pH (*c* 0.5 H_2O) 6.70. $[\alpha]_D^{20}$ (*c* 0.5 H_2O) +220.93 (°)·ml·dm^{-1}·g^{-1}. FTIR (ν/cm^{-1}): 3435, 1622, 1418, 1333, 1240, 1145, 1100, 1017, 953. Metal content (%): Na 11.35; Ag 0.52	99:1	Yield 89%. Moisture 10.6%. pH (*c* 0.5 H_2O) 6.68. $[\alpha]_D^{20}$ (*c* 0.5 H_2O) +222.0 (°)·ml·dm^{-1}·g^{-1}. FTIR (ν/cm^{-1}): 3457, 1612, 1417, 1333, 1238, 1147, 1101, 1015, 953. Metal content (%): Na 11.48; Ag 0.54
19:1	Yield 91%. Moisture 9.2%. pH (*c* 0.5 H_2O) 6.46. $[\alpha]_D^{20}$ (*c* 0.5 H_2O) +153.81 (°)·ml·dm^{-1}·g^{-1}. FTIR (ν/cm^{-1}): 3342, 1608, 1418, 1334, 1237, 1147, 1101, 1015, 952. Metal content (%): Na 10.84; Ag 2.59	19:1	Yield 92%. Moisture 10.0%. pH (*c* 0.5 H_2O) 6.38. $[\alpha]_D^{20}$ (*c* 0.5 H_2O) +185.71 (°)·ml·dm^{-1}·g^{-1}. FTIR (ν/cm^{-1}): 3430, 1617, 1419, 1333, 1240, 1147, 1099, 1015, 952. Metal content (%): Na 11.15; Ag 2.87
9:1	Yield 86%. Moisture 9.6%. pH (*c* 0.5 H_2O) 6.18. $[\alpha]_D^{20}$ (*c* 0.5 H_2O) +140.99 (°)·ml·dm^{-1}·g^{-1}. FTIR (ν/cm^{-1}): 3427, 1611, 1418, 1332, 1238, 1147, 1100, 1014, 952. Metal content (%): Na 10.37; Ag 5.13	9:1	Yield 92%. Moisture 9.4%. pH (*c* 0.5 H_2O) 6.19. $[\alpha]_D^{20}$ (*c* 0.5 H_2O) +124.67 (°)·ml·dm^{-1}·g^{-1}. FTIR (ν/cm^{-1}): 3439, 1610, 1417, 1332, 1236, 1146, 1101, 1015, 952. Metal content (%): Na 10.26; Ag 5.30

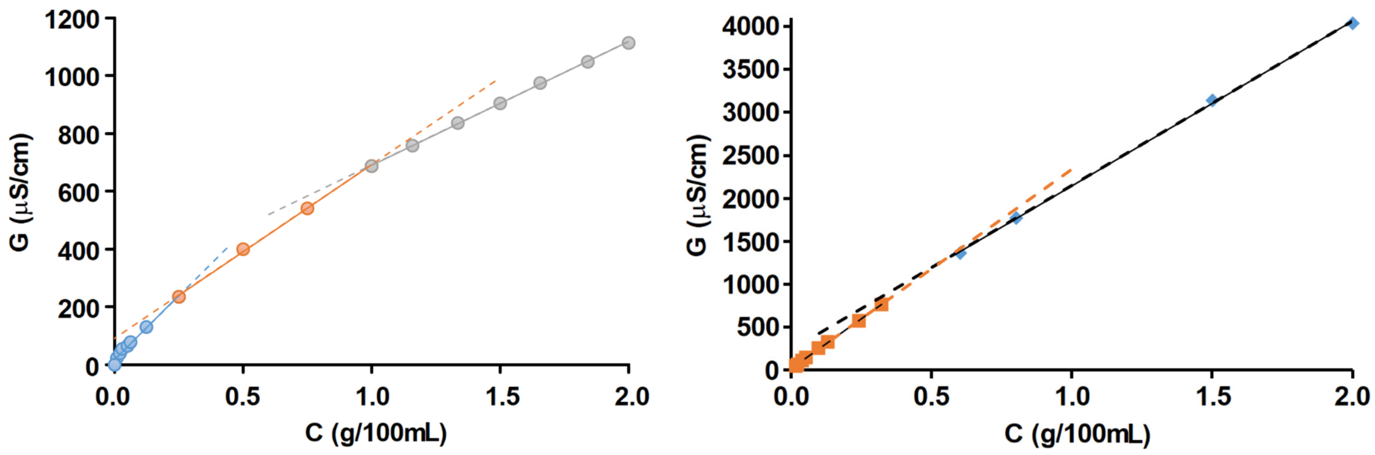

(**a**) (**b**)

Figure S1. Concentration dependences of the electroconductivity of pectin (**a**) and sodium polygalacturonate (**b**) in aqueous solution.

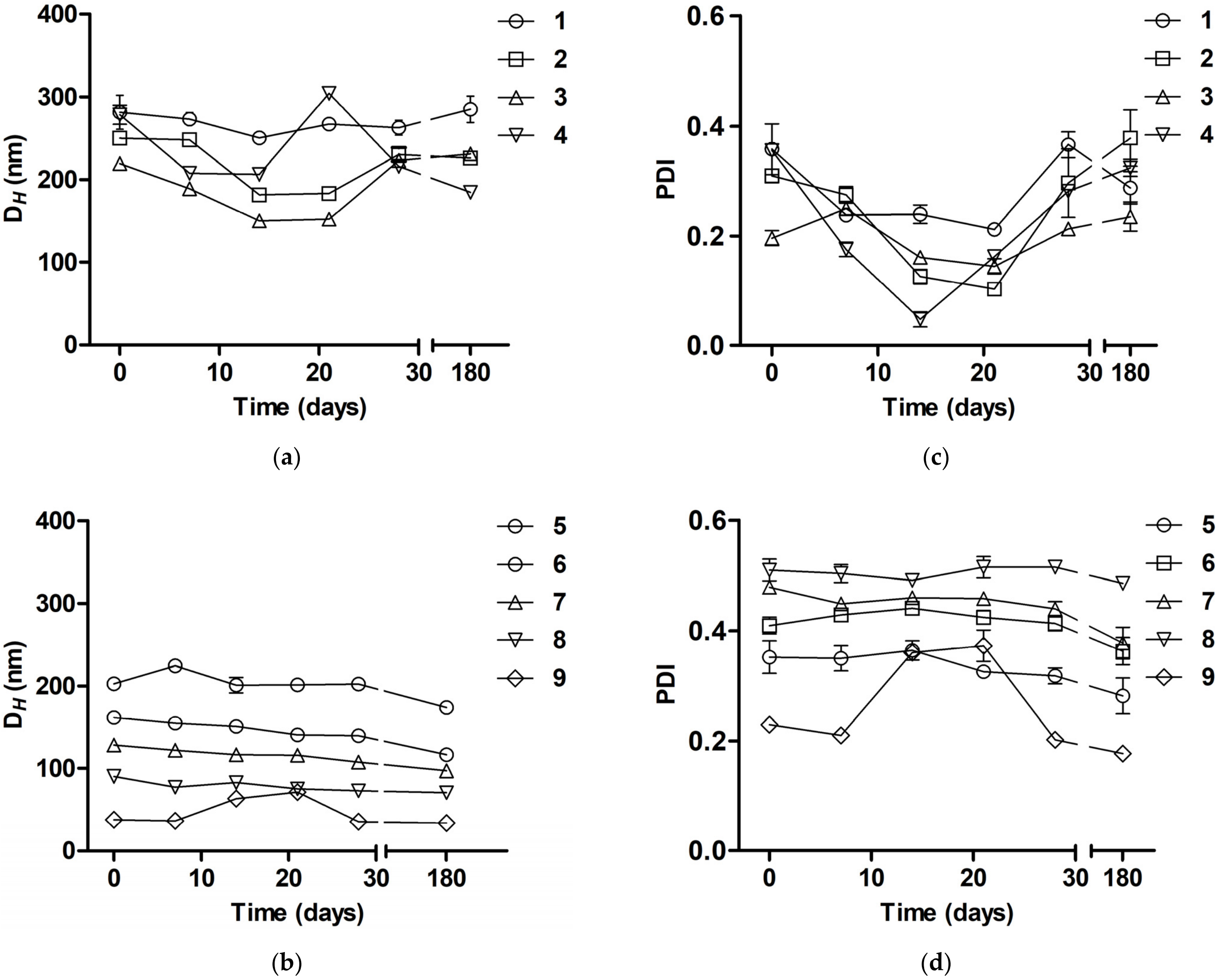

Figure S2. Changes in (**a**, **b**) hydrodynamic diameter and (**c**, **d**) particle dispersion index of silver-containing complexes upon storage in aqueous solution (1 mg/mL).

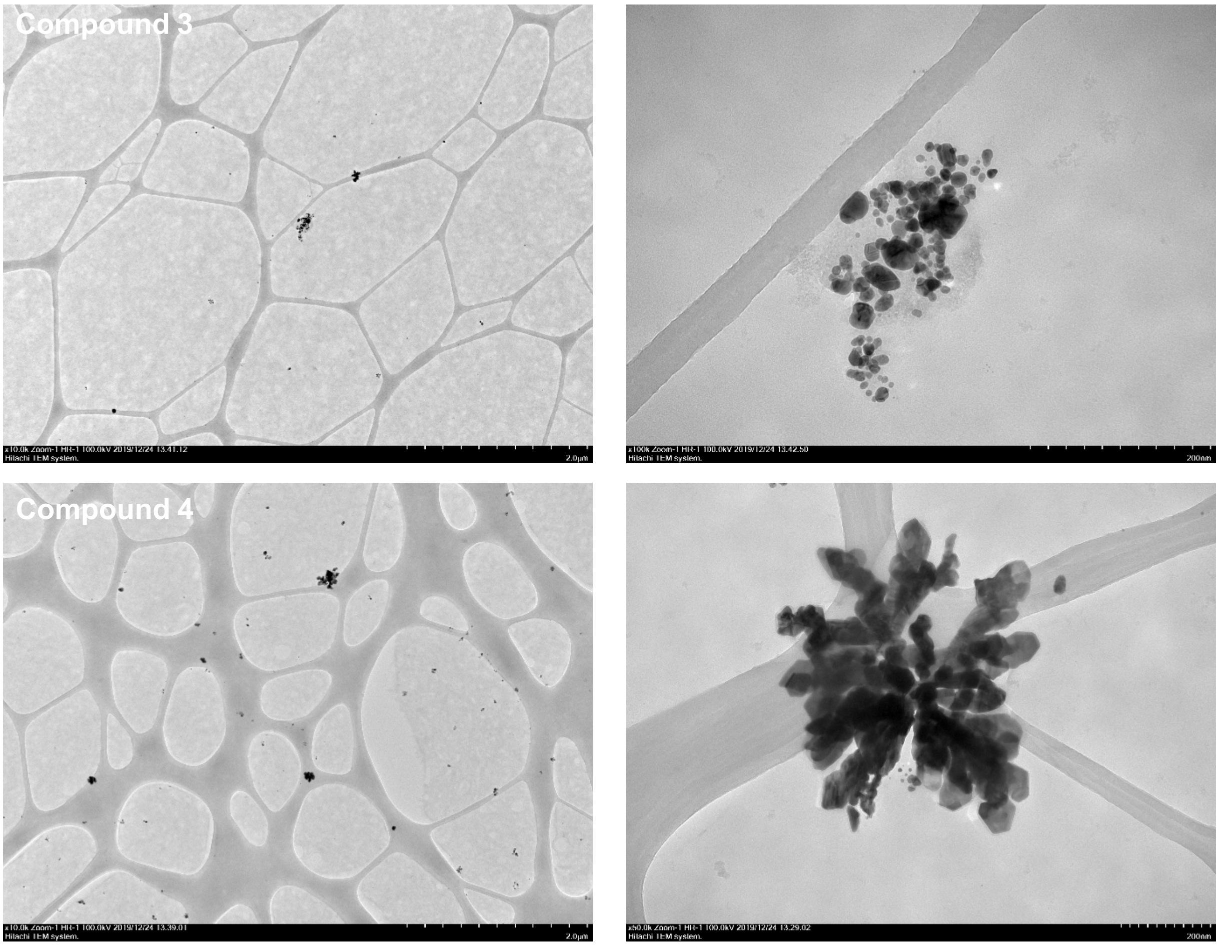

Figure S3. Representative TEM images of silver-polygalacturonate complex **3** (upper panel) and complex **4** (lower panel) at different magnifications.

Table S2. MIC values (mg/mL) of silver-containing complexes: as-prepared (left panels) and after storage for 7 days (right panels).

No	*S.a.*		*B.s.*	
**1**	2.4	2.4	2.4	2.4
**2**	1.2	1.2	0.6	1.2
**3**	0.6	0.6	0.3	0.6
**4**	0.6	0.6	0.3	0.3
**5**	2.3	2.3	2.3	2.3
**6**	1.2	1.2	0.6	1.2
**7**	0.6	0.6	0.3	0.6
**8**	0.3	0.3	0.2	0.3
**9**	0.2	0.2	0.2	0.2

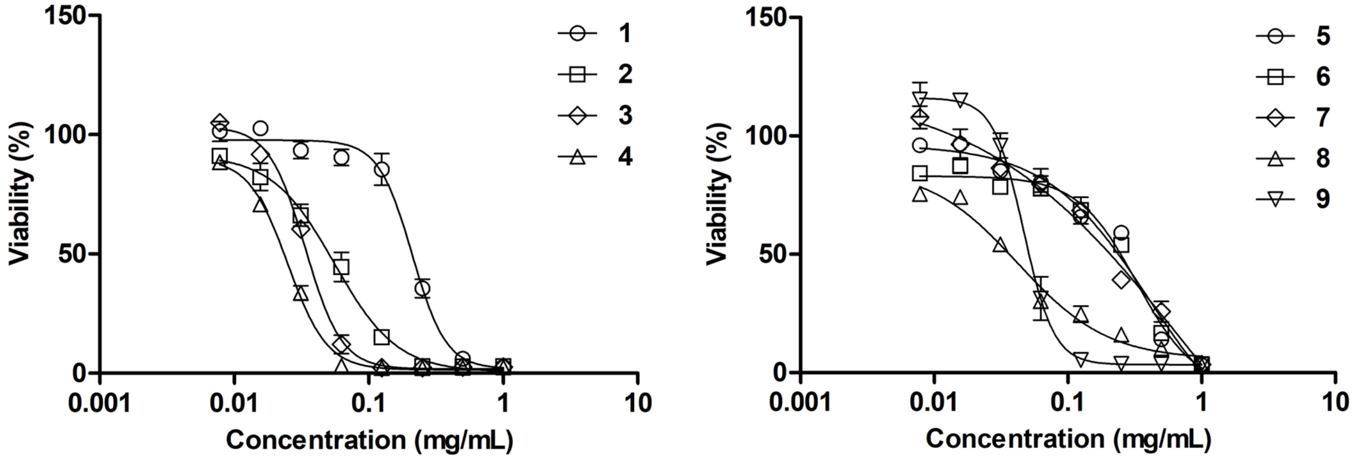

(**a**)

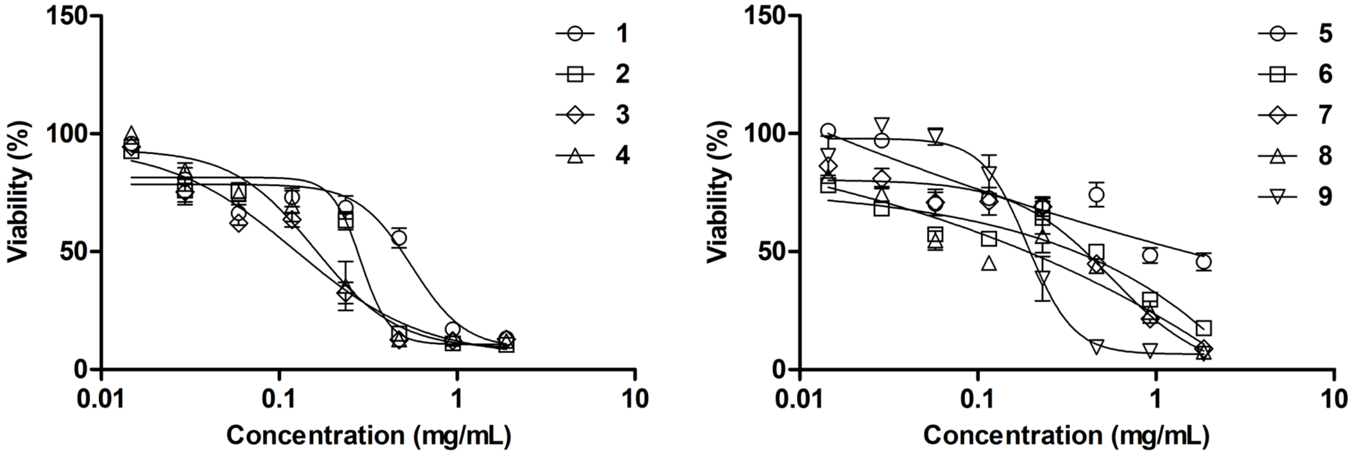

(**b**)

Figure S4. Concentration-dependent cytotoxicity of silver-containing complexes toward (**a**) 3T3 cells and (**b**) HSFs (resazurin assay, 72 h).

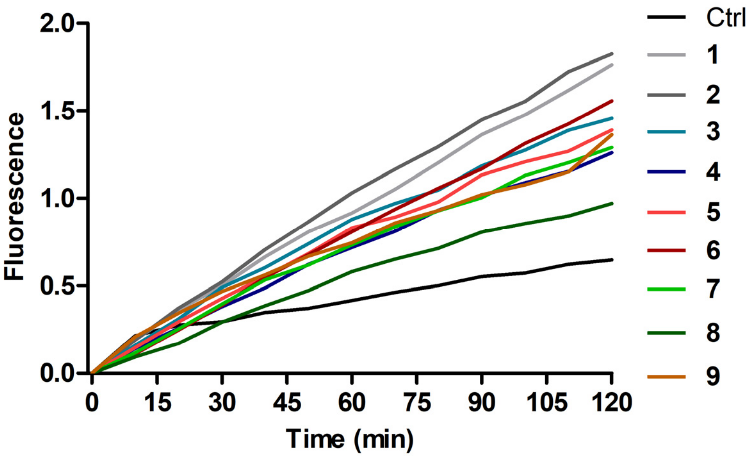

Figure S5. Effect of silver-containing complexes on ROS generation in 3T3 cells according to DCFDA fluorescence.

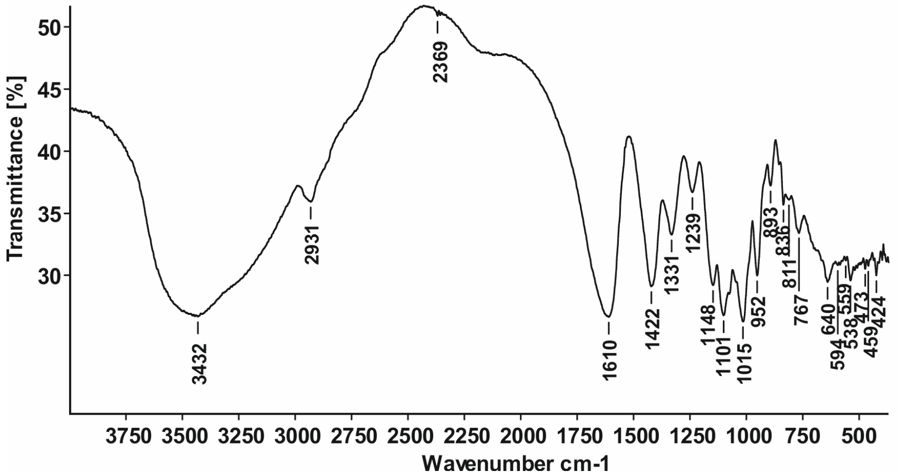

Figure S6. IR spectrum of sodium polygalacturonate.

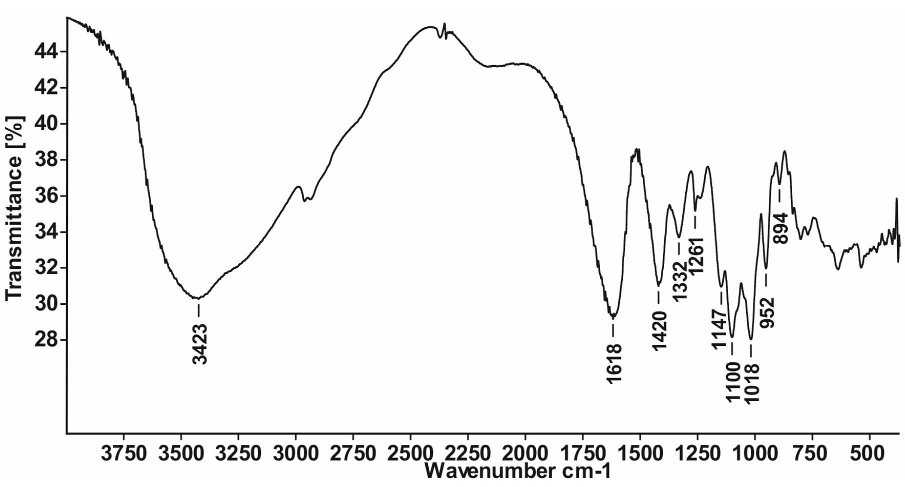

Figure S7. IR spectrum of oxidized sodium polygalacturonate (PGNa_Ox).

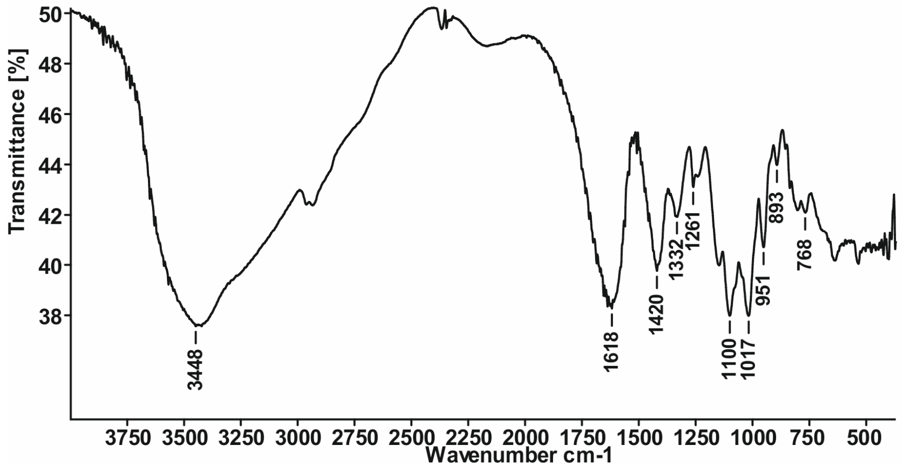

Figure S8. IR spectrum of reduced sodium polygalacturonate (PGNa_Red).

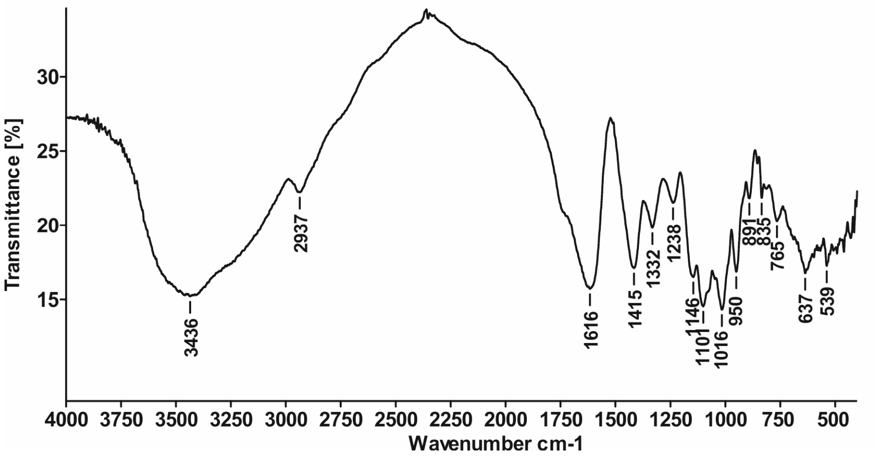

Figure S9. IR spectrum of silver polygalacturonate **1**.

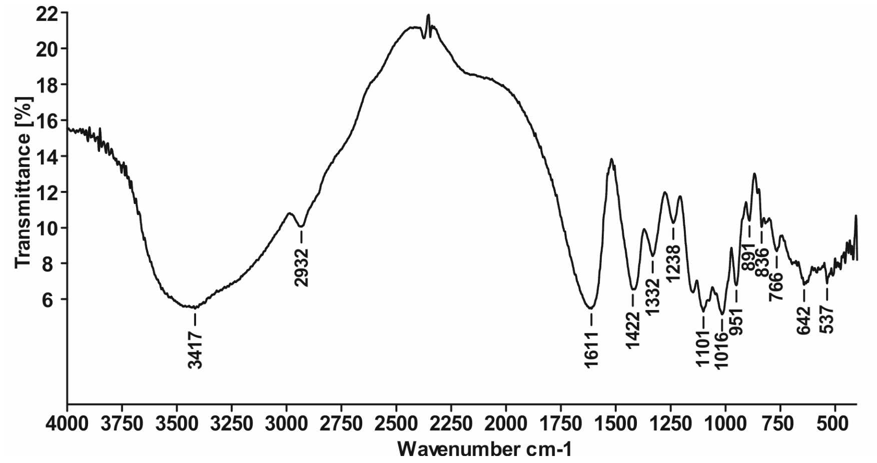

Figure S10. IR spectrum of silver polygalacturonate **2**.

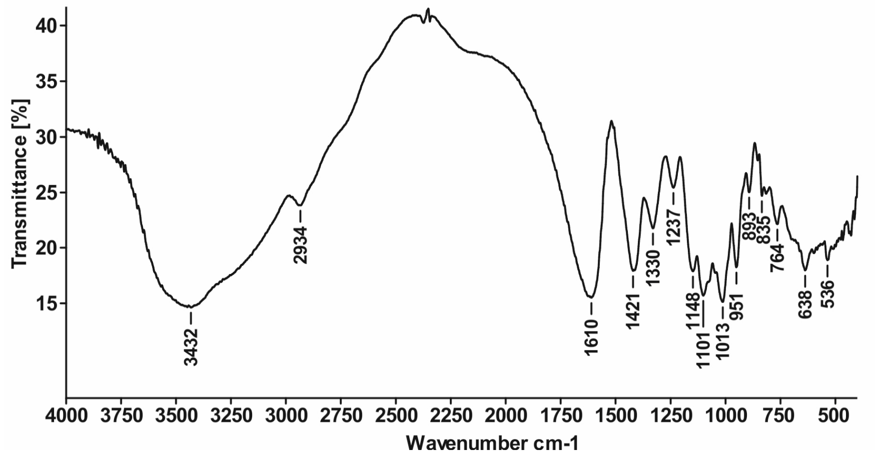

Figure S11. IR spectrum of silver polygalacturonate **3**.

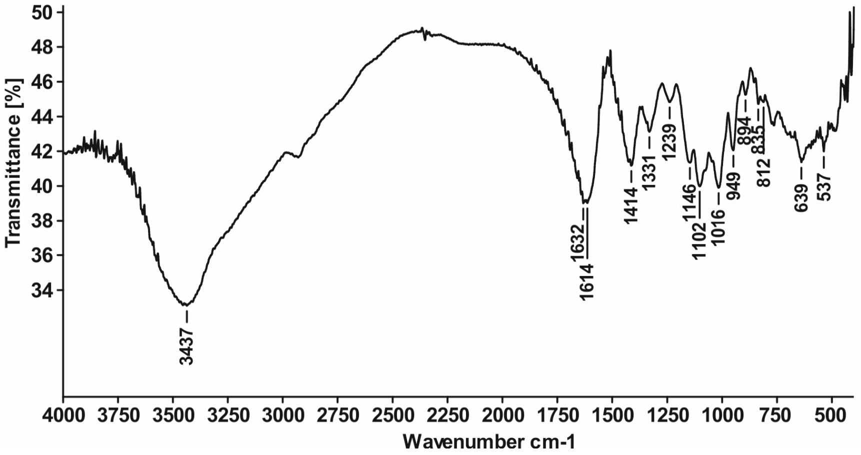

Figure S12. IR spectrum of silver polygalacturonate **4**.

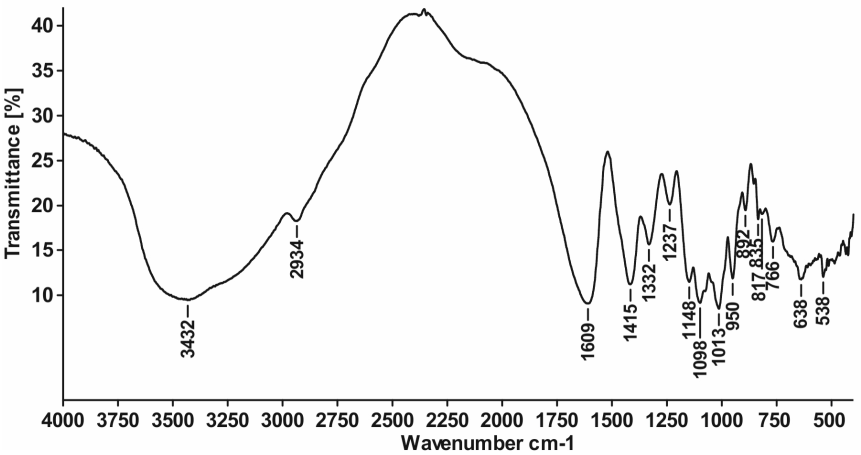

Figure S13. IR spectrum of silver polygalacturonate **5.**

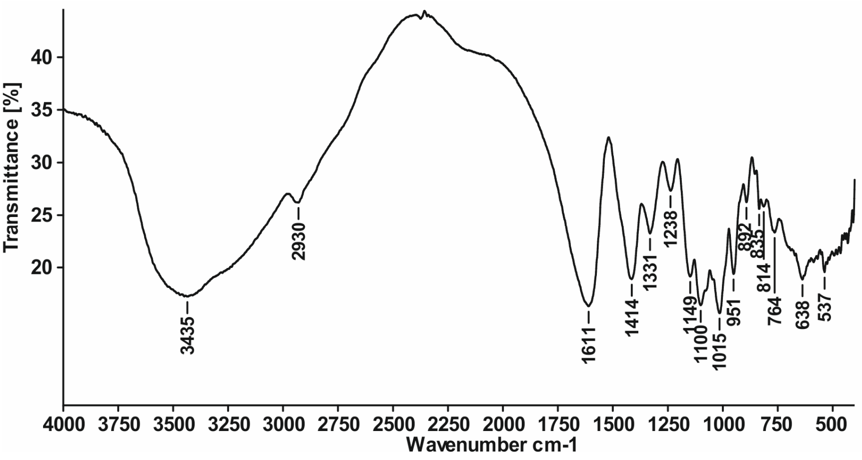

Figure S14. IR spectrum of silver polygalacturonate **6**.

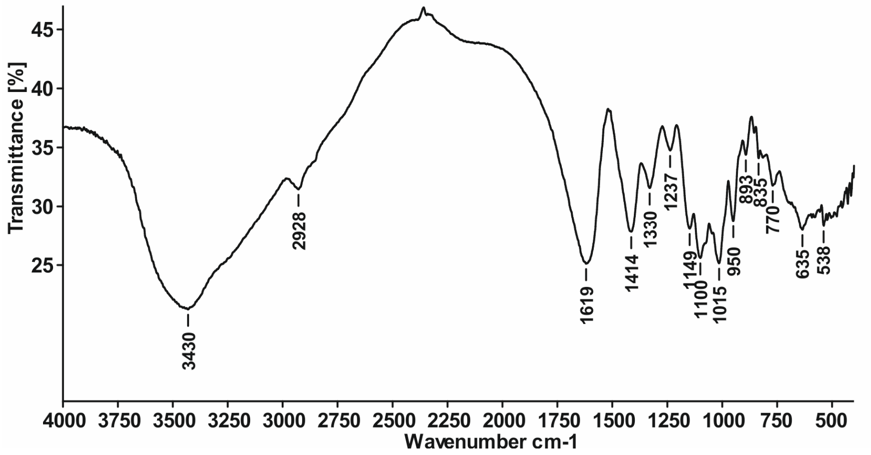

Figure S15. IR spectrum of silver polygalacturonate **7**.

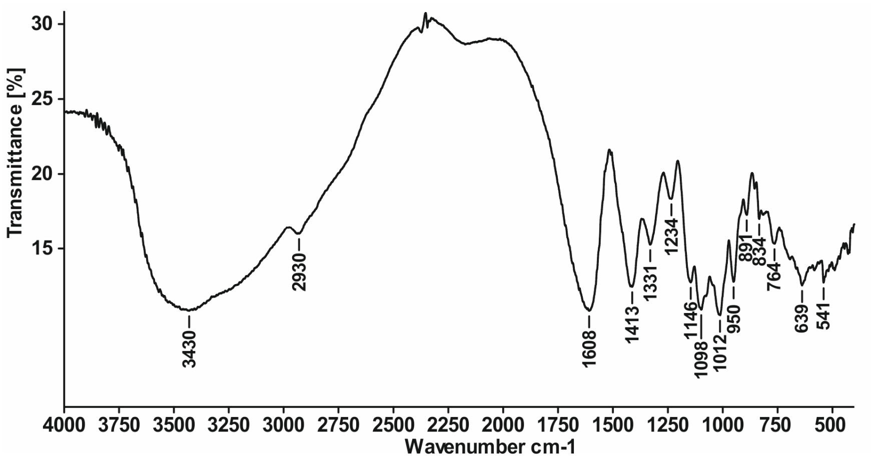

Figure S16. IR spectrum of silver polygalacturonate **8**.

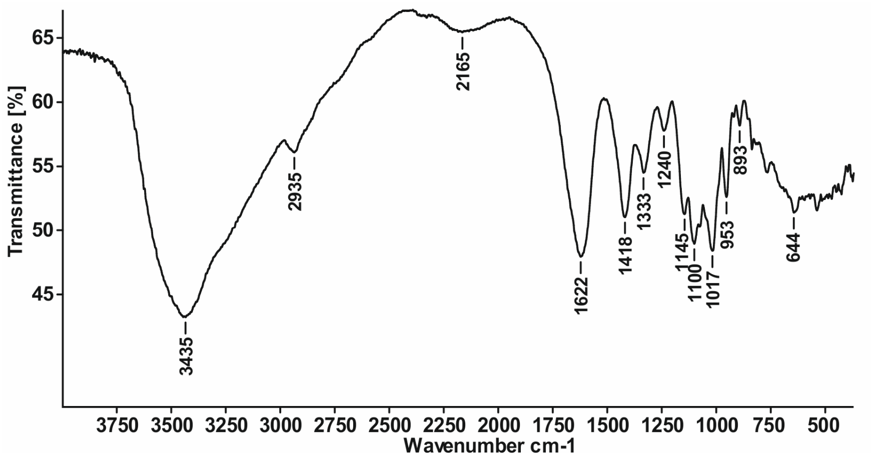

Figure S17. IR spectrum of silver-polygalacturonate complex prepared at RT (Na:Ag ratio 99:1).

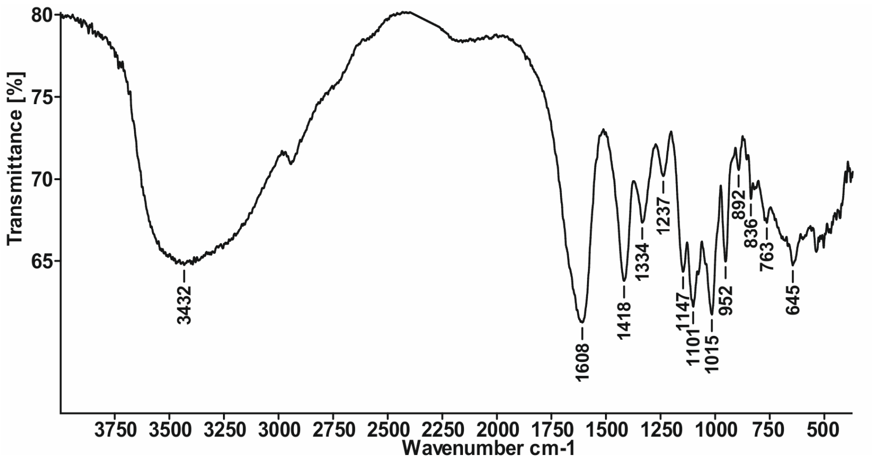

Figure S18. IR spectrum of silver-polygalacturonate complex prepared at RT (Na:Ag ratio 19:1).

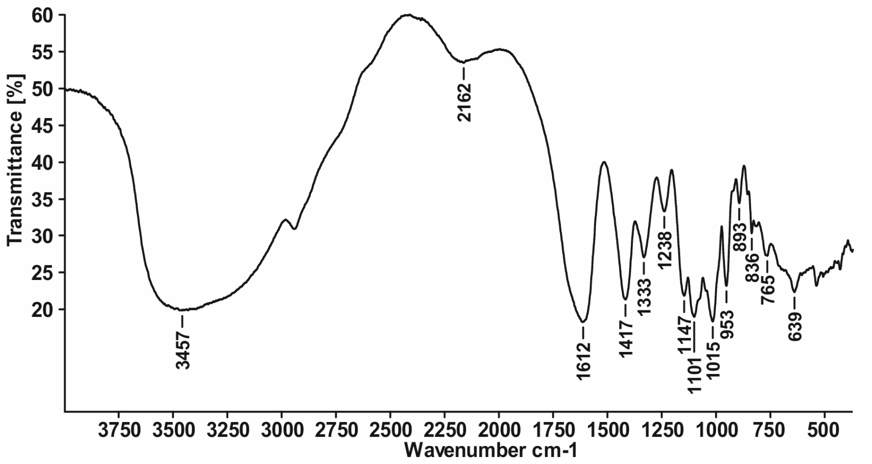

Figure S19. IR spectrum of silver-polygalacturonate complex prepared at RT (Na:Ag ratio 9:1).

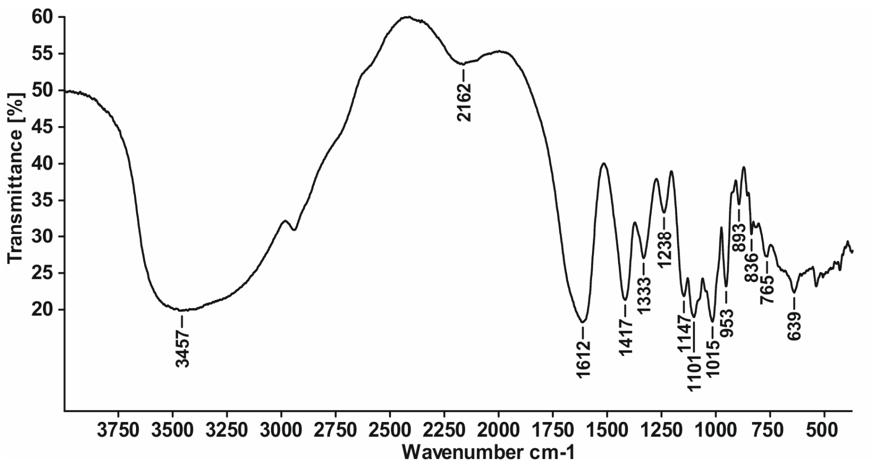

Figure S20. IR spectrum of silver-polygalacturonate complex prepared at 60 °C (Na:Ag ratio 99:1).

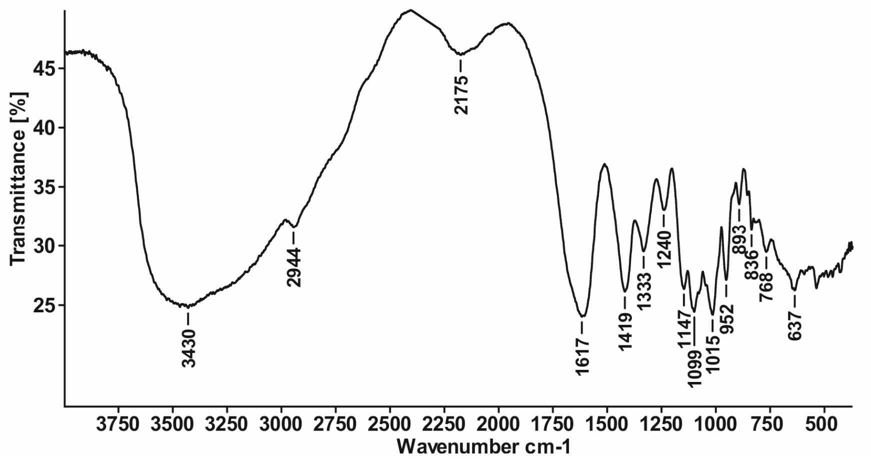

Figure S21. IR spectrum of silver-polygalacturonate complex prepared at 60 °C (Na:Ag ratio 19:1).

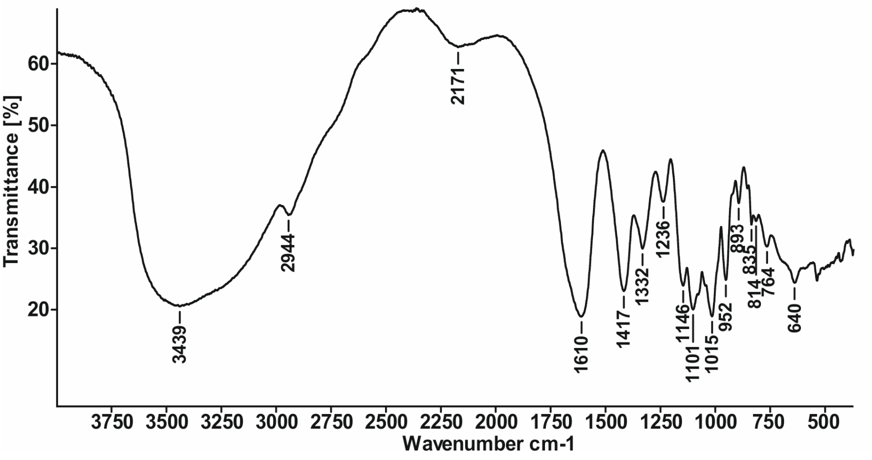

Figure S22. IR spectrum of silver-polygalacturonate complex prepared at 60 °C (Na:Ag ratio 9:1).
